# Evaluating and predicting the stickiness regime of drying droplets across drying conditions

**DOI:** 10.1016/j.crfs.2026.101588

**Published:** 2026-09-29

**Authors:** S.W.C. de Leeuw, J. Bekebrede, M.A.I. Schutyser, R.G.M. van der Sman

**Affiliations:** a Laboratory of Food Process Engineering, Wageningen University & Research, P.O. Box 17, Wageningen, 6700 AA, the Netherlands; b Food and Biobased Research, Wageningen University & Research, PO Box 17, Wageningen, 6700 AA, the Netherlands

**Keywords:** Spray drying, Agglomeration, Locking point, Glass transition, Predictive modeling, Relative humidity

## Abstract

One of the key reasons spray drying yields high-quality powders is that it promotes particle agglomeration. This occurs thanks to sticking collisions between drying droplets and/or dry fine particles. Unfortunately, the effect of drying conditions on the stickiness regime is not yet studied in detail, which makes it harder to steer agglomeration in practice. This research therefore aims to unravel the effect of relative humidity and temperature on the stickiness regime and to predict the stickiness regime across these drying conditions. A sessile single droplet drying platform was employed to perform drying and binary collision studies at different relative humidities and temperatures. Subsequently, a predictive diffusion model was verified with high-speed imaging and high-resolution thermography and used for connecting surface properties and the stickiness regime. Increased relative humidity and decreased temperature led to an increase in drying time and thus later locking point. The stickiness regime shifted with locking point at higher relative humidities but was not prolonged. Finally, the model could successfully correlate droplet surface properties (T_g_/T) to collision outcomes and could thus predict the stickiness regime based on locking point and surface properties. This approach provides a new general framework for determining the stickiness regime of single droplets.

## Highlights

• RH and temperature affect the locking point time of drying droplets.
• A predictive droplet drying model was verified with volume and temperature data.
• The stickiness regime is identified between 0.75<tcollision/tlock<1.5
• Surface properties are linked to the stickiness regime via Tg/T.
• A general approach for predicting sticking behaviour in spray drying is proposed.

## Graphical abstract

**Figure:**
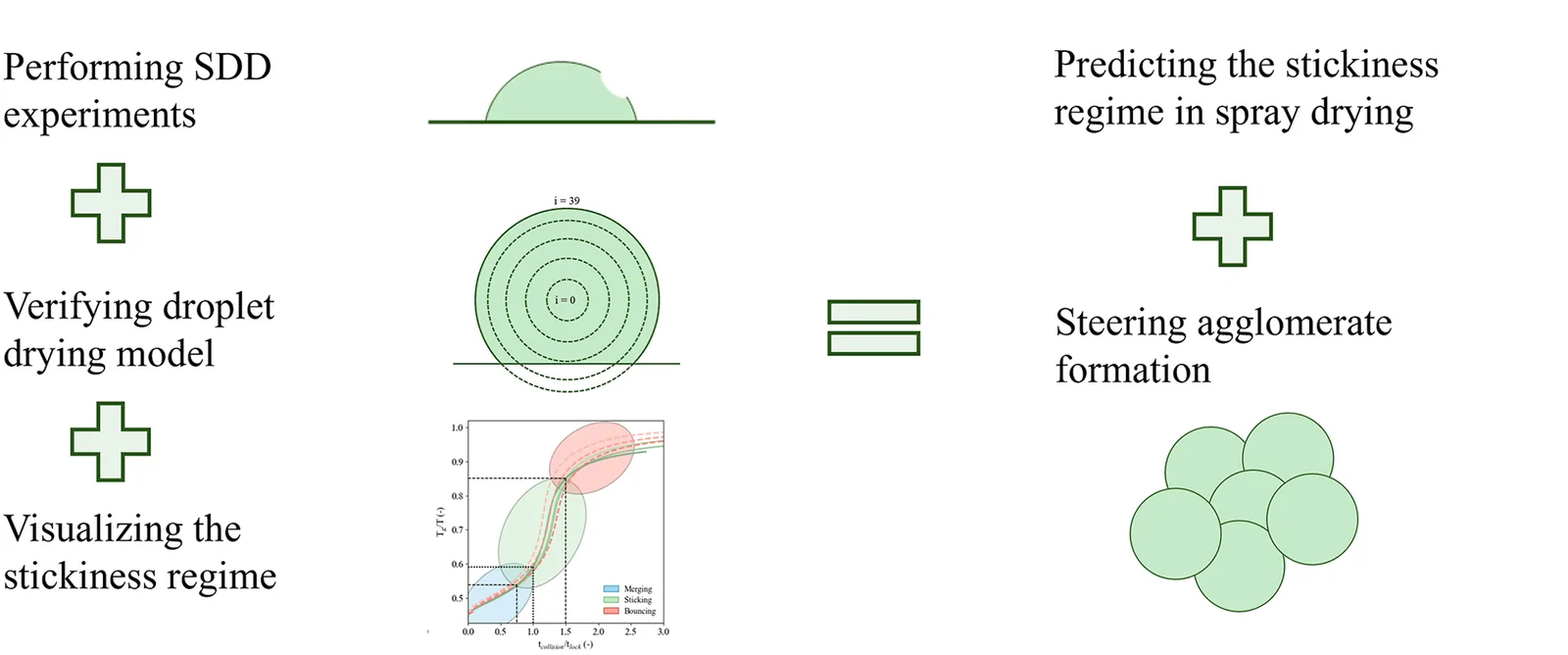

## 1 Introduction

Spray drying can provide high quality food powders with excellent techno-functional properties, especially because particles are agglomerated. Agglomerates formed during spray drying are larger porous structures consisting of multiple primary dried droplets formed through both droplet-droplet and droplet-fine collisions (Verdurmen et al., 2004). Fines are very small, dry particles (generally <100 μm) that are recirculated from the fluidized bed or cyclone into an industrial spray dryer to enhance nozzle-zone agglomeration (Gianfrancesco et al., 2008). Collisions between droplets and fines can be classified into characteristic regimes which mostly depend on the state of the drying droplet. First, a collision between a wet droplet and a fine particle results in complete merging of the particle into the droplet. Second, sticking collisions occur, when a droplet has a partially dried surface and a fine particle literally sticks to the droplet after collision (Adhikari et al., 2001). As drying proceeds, the droplet surface moves closer to glass transition, such that collisions between the drying droplet and fine particle result in bouncing (Roos, 2002). Agglomeration is a complex process, and the underlying phenomena of agglomeration have not been quantified in detail. Recent CFD work has contributed to a more quantitative understanding of droplet collisions and agglomeration, by for example identifying the sticky zone in pilot scale spray drying and predicting the final powder particle size after agglomeration (Hussain et al., 2022; Jaskulski et al., 2015; Jubaer et al., 2020). Even though heat & mass transfer, droplet shrinkage and collision rules are considered in these CFD models, particle surface stickiness in relation to agglomeration at a mechanistic level is not yet fully understood.

As mentioned above, agglomeration occurs primarily in the nozzle zone of a spray dryer (Eijkelboom et al., 2023). In this zone, the drying air phase quickly evolves from its inlet to its chamber (similar to outlet) conditions, implying that the temperature rapidly decreases to below 100 °C and the relative humidity (RH) increases from close to zero to around 10-15%. Both air temperature and relative humidity affect drying kinetics of droplets and thus the stickiness regime and resulting agglomeration behavior.

To better understand droplet drying during spray drying, different single droplet drying (SDD) approaches have been developed (Eijkelboom et al., 2023; Fu et al., 2012). SDD approaches offer detailed insights into how drying affects bacterial viability, enzyme stability, particle morphology, encapsulation and solubility across diverse food and pharmaceutical matrices at single droplet scale (Al-Khattawi et al., 2018; Sadek et al., 2015; Schiffter and Lee, 2007; Schutyser et al., 2012; Sewalt, 2024; Shamaei et al., 2016; Tran et al., 2016). Specifically, a sessile SDD approach was used for studying binary collisions between drying droplets and glass beads, acting as fines particles to better understand sticking collisions that are critical to agglomeration (Eijkelboom et al., 2024; van Boven et al., 2024). Sessile SDD with collisions could provide insights into drying kinetics, morphology development and allowed analysis of collision regimes for different formulations. Although these studies offer important insights into the stickiness regime, it remains challenging to extrapolate such findings to larger scales. This motivates the need to develop methods for predicting the stickiness regime beyond existing empirical rules.

In previous SDD studies the locking point has been defined as the start of the transition from the constant to the falling rate period, when drying becomes diffusion limited (Mezhericher et al., 2011; Shamaei et al., 2016; Tran et al., 2016). For previous *sessile* SDD studies, this so-called locking point is visually observed when a drying droplet does not retain its ideal spherical shape any longer. In line with previous observations, it is assumed that this locking point coincides roughly with the onset of skin formation. The skin properties also affect the morphology development of the drying droplets (Giorgiutti-Dauphiné and Pauchard, 2013; Siemons et al., 2020). Next to morphology development, the locking point was also related to the stickiness regime of maltodextrin droplets with varying dextrose equivalence (DE), where sticking collisions occurred at 0.75 < $\left(\frac{t_{collision}}{t_{lock}}\right)$ < 1.5 (Eijkelboom et al., 2024). Besides the locking point, the glassy state has been considered a key parameter in determining the stickiness regime. Petersen (2015) attempted to correlate drying time to the boundary of sticking to non-sticking and for skim milk droplets it was found that the time the glassy state is reached roughly scales with the square of the initial droplet diameter $\left(t_{dry}∼d_{0}^{2}\right)$. Sewalt (2024) suggested normalizing drying time with the time for the droplet surface to reach the glassy state $\left(\frac{t_{dry}}{t_{glass}}\right)$ and found that the transition from merging/sticking to bouncing could thus be roughly estimated. Glass transition temperature is highly affected by moisture content. It was found the rheological properties of polymers, such as maltodextrins, are also dependent on both moisture content and temperature and could therefore be related to the ratio of glass transition temperature over the droplet temperature (T_g_/T) (Siemons et al., 2022; van der Sman et al., 2022, 2026). It was found T_g_/T scales with viscosity of maltodextrin solutions with varying DE (Siemons et al., 2020). A steeper increase in viscosity as a function of T_g_/T was found for low DE maltodextrins, which is relevant to sticking behavior of drying droplets with different DE values. It is thus hypothesized that the ratio of T_g_/T close to the droplet surface is highly relevant to identify sticky behavior of single droplets during drying. As indicated previously, the surface properties of drying droplets can be expected to be highly dependent on local drying air conditions, such as temperature and relative humidity, and are thus important to consider when translating insights to larger scale.

This research therefore aims to unravel the impact of relative humidity and temperature of the drying air on the stickiness of single droplets and to predict the stickiness regime across different drying conditions based on particle surface conditions. This was conducted using sessile single droplet drying experiments, where the locking point was determined, and drying behavior was monitored. In addition, droplet temperature was monitored using an infrared (IR) camera and stickiness regimes were examined through binary collision experiments. Experimental findings were used to validate a droplet drying model. Last, calculated surface properties were analyzed to predict the stickiness regime for different drying conditions using a combination of experimental and modeling work. The results of this study can aid in further enhancing nozzle zone agglomeration in spray drying operations.

## 2 Materials & methods

### 2.1 Materials

Maltodextrin (MD) with DE 38 (Glucidex 38, Roquette Frères, Lestrem, France) was used as model material for drying experiments. MD is a well characterized ingredient with extensively characterized physical properties (e.g., diffusivity, sorption isotherms), which makes it highly suitable for physical modeling. MD solutions (20% (w/w)) were made with demineralized water and stirred for 30 min at 300 rpm under ambient conditions to obtain transparent and homogeneous solutions. Glass beads were used as collision particles and obtained from EPCE Bouwstoffen B.V. (the Netherlands), in the size range 70-110 μm (density of 3220 kg/m^3^). In our previous research it was concluded that collision outcomes were primarily governed by the state of the drying droplet as glass beads behaved similarly to maltodextrin fines particles and glass beads can therefore be used to mimic maltodextrin fines particles in single droplet drying studies (de Leeuw et al., 2025).

### 2.2 Methods

#### 2.2.1 Sessile single droplet drying and binary collisions

A custom-built sessile single droplet dryer (SDD) was used for dispensing and drying droplets on a surface. Droplets of 400 μm were dispensed on the drying table and dried using an air stream of 80 °C at 0.4 m/s and varying RH. With thermocouples both the air temperature and surface temperature near the droplet were recorded. Droplet drying and binary collisions were recorded using a high-speed camera (C-VIT, AOS Technologies AG, Switzerland). To determine the merging, sticking, and bouncing regime of drying droplets at different relative humidities, collision experiments were performed using an advanced sessile single droplet drying platform (Fig. 1). First, approximately 25 mg of collision particles are loaded into a dosing funnel. When triggered, the funnel can release the collision particles into the air channel, where the collision particles are dragged along with the air stream to finally reach the dispensed drying droplet. Selected collision time ranges were 1-12, 4-15 and 8-20 s, for 0%, 10% and 15% RH, respectively. These selected collision time ranges were based on earlier observations that changes in collision behavior occur mostly around the locking point (Eijkelboom et al., 2024). Approximately, 10 collision measurements per collision time point were observed. For more detailed descriptions of the experimental setup and procedures used for collision experiments during single droplet drying, we refer to Eijkelboom et al. (2024).

**Fig. 1:**
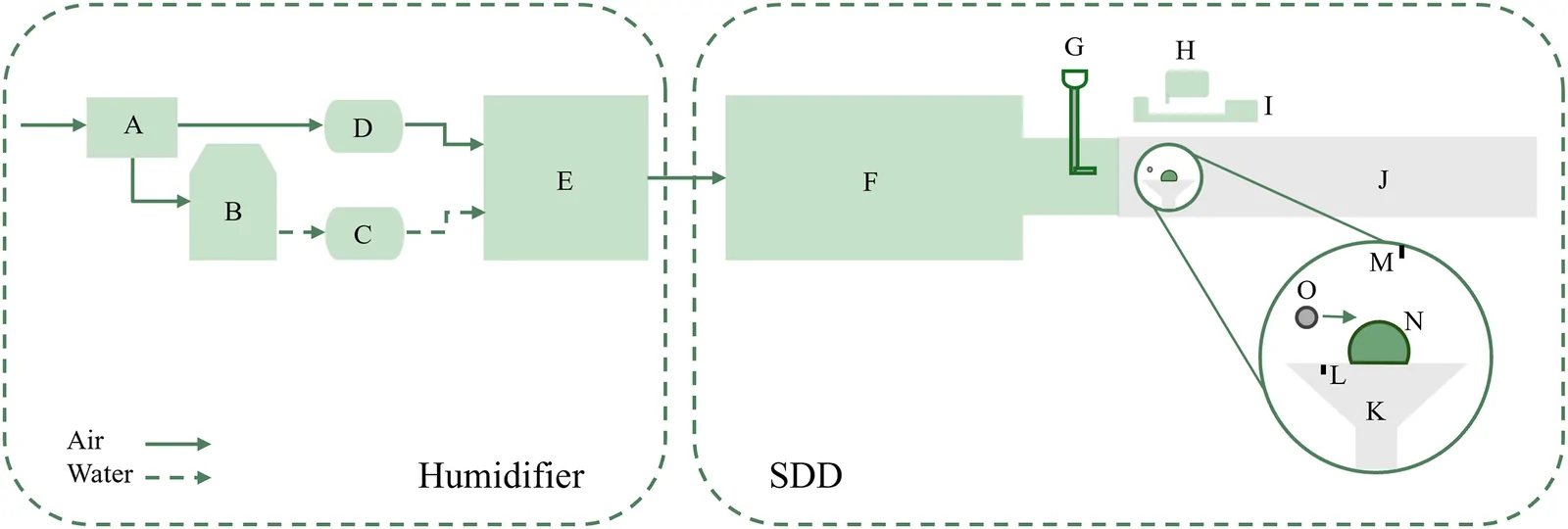
Schematic overview of sessile single droplet drying platform, extended with the humidifier. A: Filter for incoming dry air, B: Water pressure tank, C: Water flow controller, D: Air flow controller, E: Controlled evaporator mixer, F: Heating block for (re)heating drying air, G: Dry particle reservoir with tube attached, H: Droplet dispenser, I: Stroboscope, J: Drying tunnel, K: Drying table, L: Thermocouple measuring surface temperature, M: Thermocouple measuring air temperature, N: Droplet on table, O: Dry particle trajectory.

To control the relative humidity of the drying air, the air inlet of the sessile single droplet drying platform was connected to a humidifier via a well-insulated tube (Fig. 1). The humidifier consists of an air and water flow controller; a heater and temperature controller; and evaporator and mixing units (CEM, Bronkhorst Nederland B.V., the Netherlands). For part of the experiments, the drying temperature was kept constant at 80 °C, while relative humidity was varied to 0, 5, 10 and 15% RH by adjusting the liquid flow to 0, 11.6, 23.6 and 36.4 g/h, respectively, with the procedure according to Veser et al. (2025). For other experiments, the drying temperature was varied to 70, 80 and 90 °C, while the liquid flow was kept at 0 g/h.

#### 2.2.2 Monitoring droplet temperature evolution

An infrared (IR) camera (PI 640 Microscope optics, Optris, Germany) was used to monitor the evolution of the droplet temperature in time. For these measurements, a special AMTIR-1 window (Edmund optics, United States) was used to look into the drying tunnel. The optical resolution of the camera was set to 640x120 pixels at a framerate of 125 fps, and the transmissivity was set to 1. Just before each measurement, the temperature measurement of the camera was internally calibrated. The actual measurement was carried out in triplicate. The setup is identical to the one shown in the figure, except that a different camera was used. To analyze droplet temperature, a measurement point of 3x3 pixels was selected and temperature profiles were gathered for every measurement point.

#### 2.2.3 Image analysis and statistical analysis of single droplets and binary collisions

##### 2.2.3.1 Single droplet drying analysis

Movies obtained using the high-speed camera were analyzed for their locking point time, volume decrease and collision outcome. The locking point is defined as the first deviation of the spherical shape in time. It is visually determined from the high-speed movies. To do so, first, a short time range of approximately 2 s is selected, during which a clear evolution from spherical droplet to more wrinkled droplet is observed. Secondly, a crosshair is placed on the droplet to assess both sides. The locking point is marked when the two sides of the droplet are no longer symmetric, with one side beginning to flatten. Given that the videos were recorded at 100 fps, the locking point can be determined more reliably from the video itself than from a series of snapshots. For that reason, two movies can be found in the repository with underlying data for this publication.

Volume decrease was determined from the movies using MATLAB (Mathworks, USA), based on the method previously described by (Both et al., 2018). The image was converted to a binary image to ensure automatic boundary recognition in the software. Droplet boundaries were detected using adaptive grayscale thresholding and circular edge detection using the MATLAB functions im2bw and imfindcircles (Fig. 2B). Sensitivity parameters were iteratively adjusted to ensure accurate droplet contour, for this the functions imcomplement, imfill, bwareopen, and imclose were used. Specular reflections were removed by user-defined masking and inpainting (Fig. 2C). For this the functions roipoly and regionfill were used, from the MATLAB Image Processing Toolbox. Regionfill alters the grayscale of the reflection area using surrounding pixels (Fig. 2D).

**Fig. 2:**
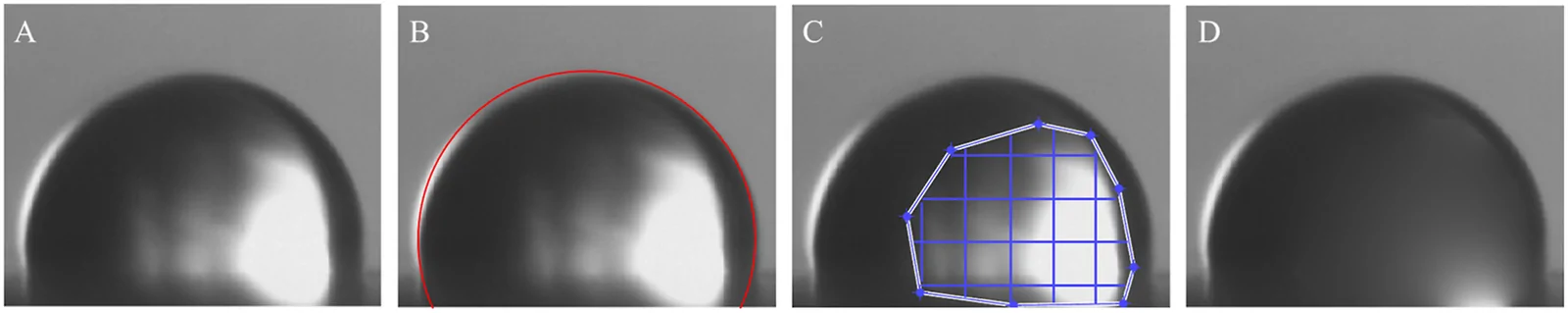
Initial droplet (A), droplet edge detection, shown in red, (B) and removal of the reflection area from the analyzed frames, using roipoly marked in blue (C) and regionfill (D).

From the detected circle and baseline, initial droplet radius, height, base diameter, and volume were computed. Additionally, the image analysis toolbox from MATLAB (MathWorks, USA) was used to invert the binary images so the droplet becomes a filled white object and the subsequent volume was determined using pixel count (Fig. 3).

**Fig. 3:**
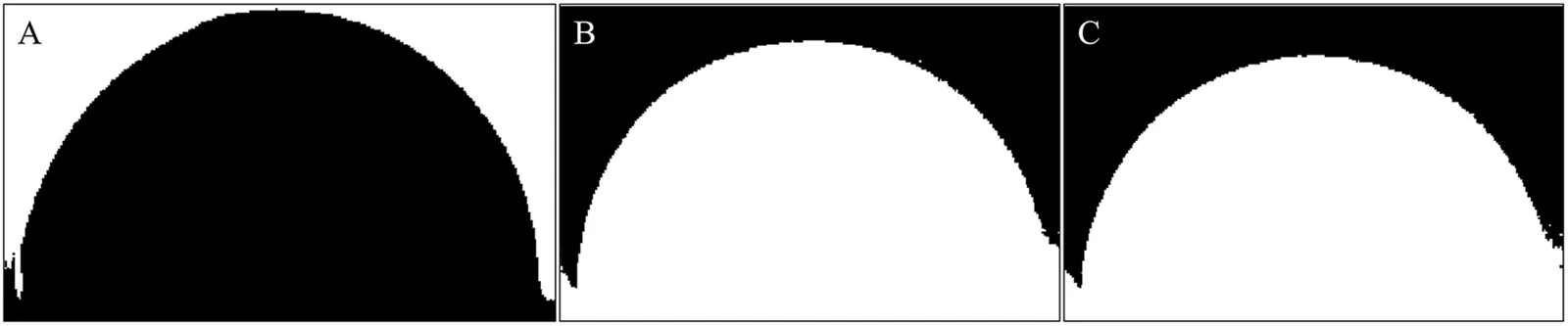
Representative example of initial droplet size recognition before inverting the binary image (A), and droplet size evolution (B, C).

Additionally, the image analysis toolbox from MATLAB (MathWorks, USA) was used to determine the droplet diameter over time, up until the locking point. A spherical cap was constructed, and the subsequent volume was determined using pixel count. The results were subsequently used to determine the volume decrease of the droplet up until the locking point time.

Lastly, statistical analysis was performed to check significant differences in the effect of RH and temperature on the normalized droplet volume at the locking point time. This was done using one-sided analysis of variance (ANOVA), a significance level of P < 0.05 was used.

##### 2.2.3.2 Collision analysis

Next, the collision outcomes were determined using the method extensively described in de Leeuw et al. (2025). Furthermore, a Gaussian kernel density estimation (KDE) was used to obtain smooth probability distributions of the collision outcomes. KDE is a non-parametric method for estimating the probability density function of a given dataset. This was done in Python using Visual Studio Code (Visual Studio Code 1.114, Microsoft, United States). This was applied twice in this research, first separately for collision outcomes for the different relative humidities. Later, the distribution of collisions was determined as a function of glass transition temperature divided by droplet temperature (T_g_/T). The KDE was implemented using the function Gaussian_kde from the SciPy library. A fixed bandwidth parameter (bw = 0.4) was applied to ensure consistent smoothing across regimes and drying conditions. The resulting density curves were normalized to their maximum value to remove the influence of unequal sample sizes, and the density profiles were smoothed using a Gaussian filter (σ = 1).

#### 2.2.4 Single droplet drying modeling

A numerical single droplet drying model was used, which was developed by Siemons et al. (2022) and adapted by Eijkelboom et al., 2023a, Eijkelboom et al., 2023b. The model is a one-dimensional predictive drying model based on heat and mass transfer theory.

First, the model uses the following differential equations for mass and heat transfer, holding in the Eularian frame.

(1) $$\begin{array}{c} \partial_{t}c_{w}+\frac{1}{r^{2}}\frac{\partial }{\partial_{r}}\left(r^{2}\cdot c_{w}\vec{u_{s}}\right)=\frac{1}{r^{2}}\frac{\partial }{\partial_{r}}\left(r^{2}\cdot D_{m}\nabla c_{w}\right) \end{array}$$

(2) $$\begin{array}{c} \partial_{t}\rho_{eff}c_{p,eff}T+\frac{1}{r^{2}}\frac{\partial }{\partial_{r}}\left(r^{2}\cdot \rho_{eff}c_{p,eff}T\vec{u_{s}}\right)=\frac{1}{r^{2}}\frac{\partial }{\partial_{r}}\left(r^{2}\cdot \lambda_{eff}\nabla T\right) \end{array}$$

Where $c_{w}$ is the mass concentration of water (kg/m^3^), $u_{i}$ is the velocity of the moving mass (m/s) (i is *w* for water and *s* for solute), $D_{m}$ is the mutual diffusivity coefficient (m^2^/s), $\rho_{eff}$ is the effective density (kg/m^3^), $c_{p,eff}$ is the effective heat capacity (J/kg·K), T is the droplet temperature (K), and $\lambda_{eff}$ is the effective heat conductivity (W/m·K). $\rho_{eff}$, $c_{p,eff}$ and $\lambda_{eff}$ are determined using the closure equations listed in Table A1 in the Appendix.

##### 2.2.4.1 Initial and boundary conditions

The initial and boundary conditions applied are the initial water concentration is uniform throughout the droplet (Eq. (3)), the initial droplet temperature is equal to the reference temperature (Eq. (4)), there is no flux of mass or heat across the center of the droplet (Eq. (5) and (6)), the diffusive flux equals the evaporative flux at the surface of the droplet (Eq. (7)), the heat transfer at the droplet-plate interface equals the conductive heat transfer (Eq. (8)) and droplet surface heat transfer is equal to the convective heat transfer and the latent heat required for evaporation (Eq. (9)).

(3) $$\begin{array}{c} c_{w\;\left(r,t=0\right)}=c_{w,0} \end{array}$$

(4) $$\begin{array}{c} T_{\left(r,t=0\right)}=T_{0} \end{array}$$

(5) $$\begin{array}{c} {\partial_{r}c}_{w\;\left(r=0\right)}=0 \end{array}$$

(6) $$\begin{array}{c} \partial_{r}T_{\left(r=0\right)}=0 \end{array}$$

(7) $$\begin{array}{c} \;-D_{m}{\partial_{r}c}_{w\;\left(r=R\right)}=j_{evap}=\beta_{ext}\left(a_{w}c_{sat}\left(T\right)-c_{air}\right) \end{array}$$

(8) $$\begin{array}{c} \;-\lambda_{eff}\partial_{r}T_{\left(surf\right)}=\frac{T_{surf}-T_{drop}}{\frac{1}{\alpha_{res}}+\frac{L_{d}}{\lambda_{d}}} \end{array}$$

(9) $$-\lambda_{eff}\partial_{r}T_{\left(r=R\right)}=h_{ext}\left(T-T_{air}\right)-\left[\Delta H_{evap,0}+\left(c_{p,v}-c_{p,w}\right)\left(T-T_{0}\right)\right]{\;j}_{evap}\;$$

Where r is the radial coordinate (m), R is the droplet radius (m), $c_{w,0}$ is the initial water concentration (kg/m^3^), $\beta_{ext}$ is the external mass transfer coefficient (m/s), $a_{w}$ is the water activity (−), $c_{sat}\left(T\right)$ is the saturated water vapor concentration at temperature T (kg/m^3^), $c_{air}$ is the water vapor concentration of the air (kg/m^3^), $T_{surf}$ is the surface temperature as experimentally measured (K), $T_{drop}$ is the droplet temperature (K), $h_{ext}$ is the external heat transfer coefficient (W/m^2^·K), $\Delta H_{evap,0}$ is the heat of vaporization of water at 273 K (J/kg), $c_{p,v}$ is the specific heat capacity of vapor (J/kg·K) and $c_{p,w}$ is the specific heat capacity of water (J/kg·K). The denominator of Eq. (8) $\left(\frac{1}{\alpha_{res}}+\frac{L_{d}}{\lambda_{d}}\right)$ will be explained more thoroughly in section 2.2.4.3.

##### 2.2.4.2 Mass transfer

Mass transfer was calculated based on differential equations. The equations were solved in a co-moving Lagrangian reference frame using the convective derivative $D_{t}=\partial_{t}+\nabla \cdot \vec{u_{s}}$ , which led to equations in the form of Fick's law (Eq. (10)).

(10) $$\begin{array}{c} D_{t}c_{w}=\nabla \cdot D_{m}\nabla c_{w\;} \end{array}$$

The evaporative mass flux $J_{\text{evap}}$ (kg/s) is determined as follows:

(11) $$\begin{array}{c} J_{\text{evap}}=f_{cap}4\pi R^{2}\beta_{ext}M_{w}\frac{p_{0}}{R_{gas}T_{air}}\ln\left(\frac{p_{vap}}{p_{0}-a_{w}p_{sat}\left(T\right)}\right) \end{array}$$

(12) $$\begin{array}{c} f_{cap}=2\pi R(\frac{r\left(1+\cos\left(\delta \right)\right)}{4\pi R^{2}})\; \end{array}$$

Where $M_{w}$ is the molecular weight of water (kg/mol), $p_{0}$ is the atmospheric pressure (Pa), $p_{vap}$ is the vapor pressure (Pa), $T_{air}$ is the air temperature (K), and $p_{sat}\left(T\right)$ is the saturated vapor pressure at air temperature T (Pa), $f_{cap}$ is a geometric factor correcting for the fact the sessile droplet has the shape of a spherical cap, R is the droplet radius and δ is the changing wall angle (rad) of the droplet.

##### 2.2.4.3 Heat transfer

The droplet temperature is assumed spatially uniform throughout drying as the Biot number, Bi ≪1. This dimensionless number represents the ratio of external and internal heat flux.

(13) $$\begin{array}{c} Bi=\frac{h_{ext}R}{\lambda_{eff}} \end{array}$$

Where $h_{ext}$ is the external heat transfer coefficient (W/m^2^·K) and $\lambda_{eff}$ is the effective thermal conductivity (W/m · K), calculated using

(14) $$\begin{array}{c} \lambda_{eff}=\varphi_{w}\lambda_{w}+\varphi_{s}\lambda_{s} \end{array}$$

Where $\lambda_{i}$ is the thermal conductivity (W/m·K), $\varphi_{i}$ is the volume fraction (−), and for both i is w for water and s for solute.

Given Bi ≪1, temperature gradients in the droplet can be safely neglected, and the Fourier or heat transfer equation can be integrated over the droplet volume (Adhikari et al., 2005). Since Bi > 0.1, thermal resistances are accounted for when describing heat transfer, using the effective heat transfer coefficient $\left(h_{eff}\right)$ and droplet internal conductive resistance $\left(\frac{L_{d}}{\lambda_{d}}\right)$ and the droplet-substrate contact resistance $\left(\frac{1}{\alpha_{res}}\right)$ (Eq. (15)) (Van Der Sman, 2003).

The equation was solved using the convective derivative $D_{t}=\partial_{t}+\nabla \cdot \vec{u_{s}}$ , which led to equations in the form of Fourier's law. Fourier's law was integrated over the volume and transformed into a single, solvable first order differential equation by applying the Gauss theorem (Whittaker, 1935). Also, the boundary condition was implemented, which led to Eq. (15) for determining the heat transfer.

(15) $$\begin{array}{c} \rho_{eff}c_{p,eff}V\frac{dT}{dt}=(h_{eff}\left(T-T_{air}\right)-\\ \left(\left[\Delta H_{evap,0}+\left(c_{p,v}-c_{p,w}\right)\left(T-T_{0}\right)\right]{\;j}_{evap}\right)A_{drop}+\left(\frac{T_{surf}-T_{drop}}{\frac{1}{\alpha_{res}}+\frac{L_{d}}{\lambda_{d}}}\right)A_{contact} \end{array}$$

Where $h_{eff}$ is the effective heat transfer coefficient (W/m^2^ · K), $T_{air}$ is the air temperature as measured close to the droplet (K). $h_{eff}$ is determined using the closure equation listed in Table A1 in the Appendix.

The conductive heat transfer from the surface to the droplet is formulated as follows:

(16) $$\begin{array}{c} F_{conduction}=A_{contact}\left(\frac{T_{surf}-T_{drop}}{\frac{1}{\alpha_{res}}+\frac{L_{d}}{\lambda_{d}}}\right) \end{array}$$

Where $A_{contact}$ is the contact area between the droplet and the surface (m^2^). Compared to previous model versions (Eijkelboom et al., 2024), the resistance term in the denominator of the equation was simplified. Since the contact surface and its characteristics are not well defined, this is captured in one parameter $\frac{1}{\alpha_{res}}$ and assumed to be 0.5-2.0∙10^−4^ (Bendada et al., 2004).

Also, the droplet internal conduction $\left(\frac{L_{d}}{\lambda_{eff}}\right)$ was added, with $L_{d}$ a characteristic length equal to $\frac{R}{5}=40\cdot 10^{-6}$ (m) and $\lambda_{eff}$ the droplet's effective thermal conductivity (W/m·K).

The contact area between the droplet and the plate ($A_{contact}$) was no longer constant but computed from the spherical cap volume calculation and updated every timestep, using equations (17) and (18).

(17) $$\begin{array}{c} R_{cap}=({\frac{3V_{drop}\;\sin^{3}\left(\theta \right)}{\pi (2-3\;\cos\left(\theta \right)+\cos^{3}\left(\theta \right)})}^{\frac{1}{3}} \end{array}$$

(18) $$\begin{array}{c} A_{contact}=\pi R_{cap}^{2} \end{array}$$

The model solves the coupled mass and energy balance equations using spherical coordinates. Also, the droplet is discretized into 40 concentric spherical shells (Fig. 4), and the governing equations are solved using a cell-centered finite volume method. Time integration is performed using a simple Euler forward scheme. Last, the model is solved in a Lagrangian reference frame that is linked to the solid material of the droplet. This means only water is allowed to diffuse between control volumes, while the amount of solids remains constant. As water evaporates, the radius, volume and position of the spherical shells are updated every timestep (dt = 0.001). These calculations are explained more elaborately in section 2.2.4.4. Based on these values, the new diffusive mass and heat fluxes are calculated.

##### 2.2.4.4 Model calculations

For $R_{i}<R-h_{cap}$ the surface area of shell (i) equals $A_{i}=4\pi r_{i}^{2}$ (m^2^). For $R_{i}>R-h_{cap}$ the surface area of shell (i) (m^2^) is calculated using Eq. 19–21. An illustration of equal volume elements, with shells i and geometry of the sessile single droplet is depicted in Fig. 4.

(19) $$\begin{array}{c} A_{i}=4\pi R^{2}-2\pi Rh_{cap}=2\pi RH_{drop} \end{array}$$

(20) $$\begin{array}{c} H_{drop}=r\left(1+\cos\left(\delta \right)\right) \end{array}$$

(21) $$\begin{array}{c} h_{cap}=r\left(1-\cos\left(\delta \right)\right) \end{array}$$

With $H_{drop}$ the height of the droplet (m), $h_{cap}$ the height of the cap (m) and δ the wall angle, being π−θ (rad).

**Fig. 4:**
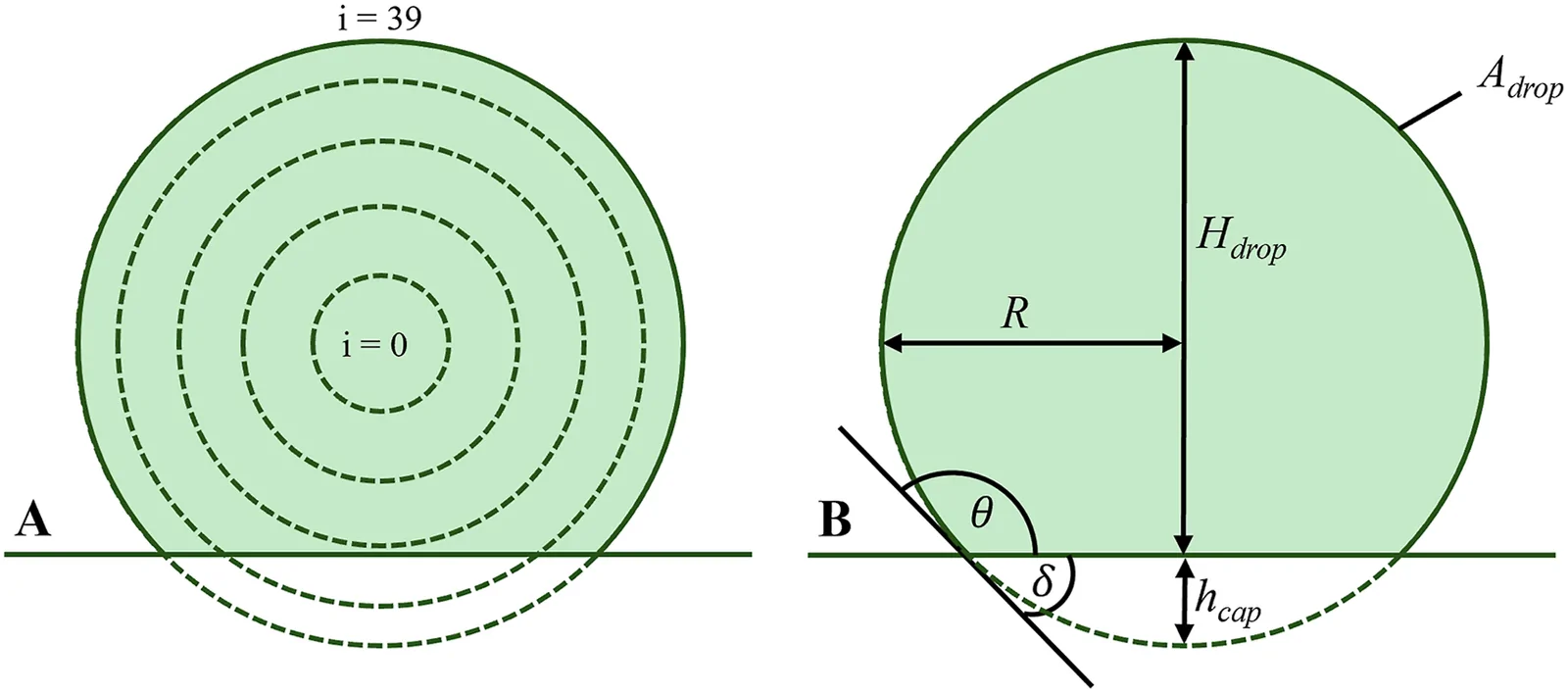
Sketch of equal volume elements, with shells i (A) and geometry of the sessile single droplet, as a spherical cap with surface area A_drop_, droplet height H_drop_, droplet radius R, cap height h_cap_, and wall angle of droplet θ and cap δ (B).

The volume of each shell $V_{i}$ (m^3^) is calculated each time step using:

(22) $$\begin{array}{c} V_{i}=\frac{m_{w,i}+m_{s,i}}{\rho_{mix}} \end{array}$$

With $m_{w,i}$ the mass of water (kg), $m_{s,i}$ the mass of solute (kg), which is constant throughout the drying process, and $\rho_{mix}$ the density of the mixture (kg/m^3^) which is based on the mass fraction of water and solute at each time step.

Within the model the glass transition temperature is predicted using the Couchman-Karasz model, which estimates $T_{g}$ as a function of moisture content (Couchman and Karasz, 1978).

(23) $$\begin{array}{c} T_{g}=\frac{y_{w}{\Delta c}_{p,w}T_{g,w}+y_{s}{\Delta c}_{p,s}T_{g,s}}{y_{w}{\Delta c}_{p,w}+y_{s}{\Delta c}_{p,w}} \end{array}$$

Where $y_{w}$ and $y_{s}$ are the mass fraction of water and solute, $T_{g,w}$ and $T_{g,s}$ are the glass transition temperatures (K) of the pure components water and maltodextrin DE38, ${\Delta c}_{p,w}$ and ${\Delta c}_{p,s}$ are the changes in specific heat at the glass transition (${\Delta c}_{p,w}$ = 1.91 kJ/kg·K, ${\Delta c}_{p,s}$ = 0.42 kJ/kg·K) (Van Der Sman and Meinders, 2011, 2013). To calculate $T_{g}$ near the surface of the droplet, the average mass fraction of water of the outer 8 (N/5 = 40/5) shells was used.

The glass transition of maltodextrin, $T_{g,s}$, can be estimated using the Flory-Fox relation (Fox and Flory, 1950; Y. Roos and Karel, 1991; Van Der Sman and Meinders, 2013).

(24) $$\begin{array}{c} T_{g,s}=T_{g,s}^{\infty }-\frac{a_{FF}}{M} \end{array}$$

$T_{g,s}^{\infty }$ is the glass transition temperature for a polymer with infinite chain length ($T_{g,s}^{\infty }$ = 475 K). $a_{FF}$ = 5∙10^4^ K·g/mol and M is the average molecular weight, calculated as $M=100*\left(\frac{180}{DE}\right)$.

##### 2.2.4.5 Calculating local relative humidity

Since, in this work the effect of relative humidity was studied, the model calculated the relative humidity close to the droplet depending on the local temperature. Antoine's equation was used to determine the saturated vapor pressure $p_{sat}$ (Pa).

(25) $$\begin{array}{c} \log_{10}\left(p_{sat}\right)=A-\frac{B}{T_{air}+C} \end{array}$$

With A, B and C being 8.07131, 1730.63 and 233.426 (−), respectively, and $T_{air}$ is the air temperature as measured close to the droplet. Next, the relative humidity, as experienced by the droplet was computed.

(26) $$\begin{array}{c} RH=\frac{x_{w}*p_{tot}}{p_{sat}\left(T_{air}\right)}=\frac{p_{vap}}{p_{sat}\left(T_{air}\right)} \end{array}$$

Where $x_{w}$ is the mole fraction of water in the air (−), which was controlled during the single droplet drying experiments (Section 2.2.1), and $p_{tot}$ is the total pressure, which is 101325 Pa. The exact RH determined at every time point can be found in Figure A1 in the Appendix.

Lastly, the closure equations, material specific parameters and experimental parameters used for solving the model can be found in Table A1 in the Appendix. The model was implemented in Python (Python 3.13.5, Python Software Foundation, United States) using the program Spyder (Spyder 6.0.7, Spyder Project Contributors, United States). As explained in section 2.2.1, the air and surface temperature near the droplet were recorded approximately every 0.2 s. For 0, 5, 10 & 15% RH and a constant temperature of 80 °C, an average of 12 measurements (3 for each scenario) were collected. For drying air temperatures of 70 and 90 °C with 0% RH, an average of 3 measurements were collected per scenario. These data were used as input for the single droplet drying model. Volume data resulting from the analysis of the single droplet drying movies, as explained in section 2.2.3, were used to fit volume reduction over time using the least squares approach. The parameter $\alpha_{res}$, which is used to determine conductive heat flux, was adjusted iteratively within a physically reasonable range.

## 3 Results & discussion

### 3.1 Drying of single maltodextrin droplets with varying RH and temperature

Single droplets of maltodextrin solutions with DE 38 were dried at varying relative humidities and temperatures, to study the effect of drying conditions on the drying kinetics of single droplets. A critical parameter in understanding drying behavior of single droplets is its locking point (de Leeuw et al., 2025; Eijkelboom et al., 2023a). Due to fast evaporation and slow solute diffusion, a steep concentration gradient evolves in the drying droplet, and close to the droplet surface this induces skin formation. This occurs when the solute concentration is sufficiently high to affect the mechanical properties of this outer part of the droplet and may thus lead to buckling. Both relative humidity and temperature influence the moment of skin locking and subsequently buckling of the droplet skin during drying. Fig. 5A illustrates a linear increase in locking point from around 6.6 to 11.8 s when increasing relative humidity from 0 to 15% at a constant temperature of 80 °C, on the other hand Fig. 5B shows a linear decrease in locking point time from around 9.4 to 5.1 s when drying air temperatures are increased from 70 °C to 90 °C at a constant RH of 0%.

**Fig. 5:**
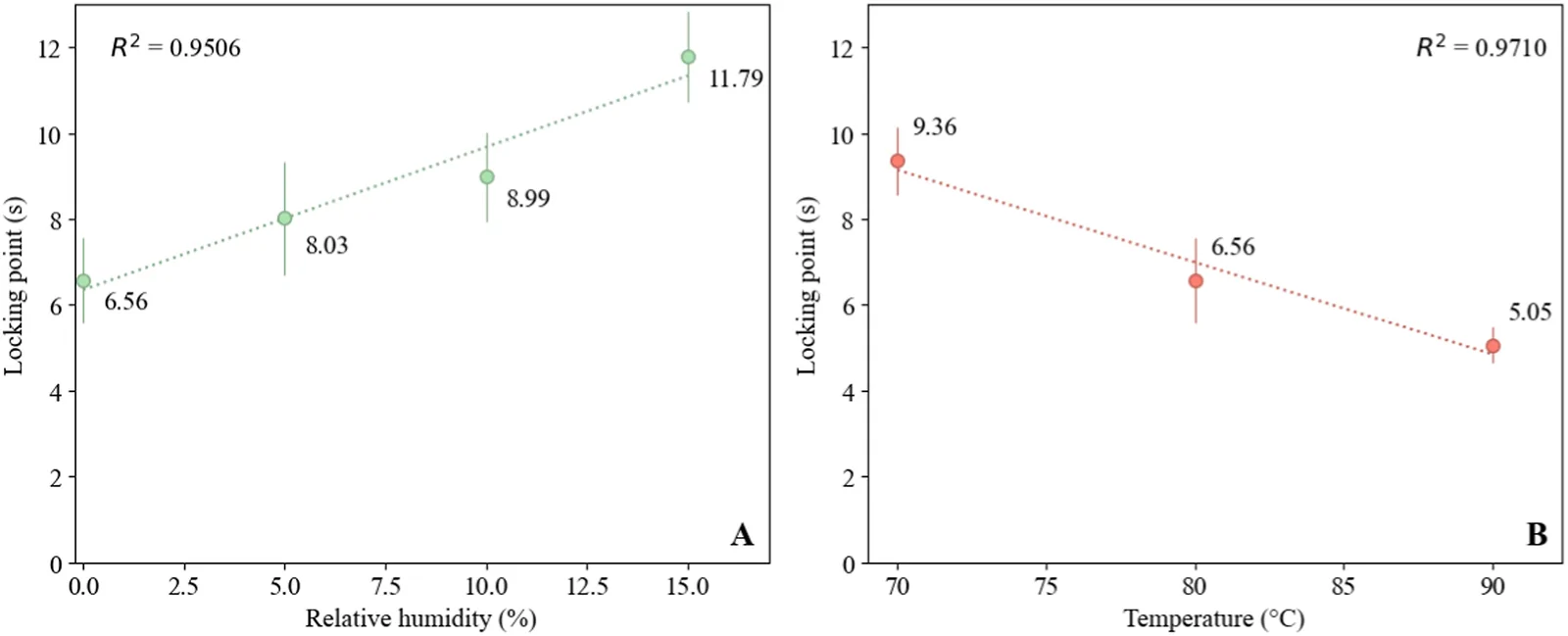
Locking point time for drying at different relative humidities (A) and temperatures (B) of maltodextrin DE38, with the 95% confidence interval given.

Before the locking point is reached the drying rate of the droplet is assumed to be constant, which is also referred to as the constant rate period (Boel et al., 2020). During this stage, droplets exhibit ideal shrinkage as water removed leads to a corresponding volume decrease. The drying rate during this phase is externally determined and is proportional to the difference in vapor pressure between the droplet and the surrounding air. Therefore, it is expected that the increase or decrease of the locking point time is proportional to the change in RH or temperature of the drying air (Tsapis et al., 2005). An increased relative humidity in the air leads to lower driving force for drying, such that the drying proceeds slower, which was also observed by Grosshans et al. (2016) when studying drying of mannitol-water droplets. Conversely, increased air temperatures lead to faster drying and so earlier locking points, which was also observed by Both et al. (2018) and Tran et al. (2016), who varied drying air temperature from 40 to 90 °C and 60-180 °C, respectively.

Given ideal shrinkage until the locking point, the radius of the drying droplets can be closely monitored and analyzed. The normalized radius (R_lock_/R_0_) at the locking point time was determined for each tested relative humidity and drying temperature (Table 1).

**Table 1:** Normalized droplet radius at the locking point time (R_lock_/R_0_) for drying at different relative humidities at T = 80 °C and different temperatures at RH = 0%. The 95% confidence interval is given. a: p < 0.05, b: p < 0.05

Drying conditions	$\frac{R_{lock}}{R_{0}}$
80 °C – 5% RH	0.842 ± 0.0163^a^
80 °C – 10% RH	0.861 ± 0.0281^a^
80 °C – 15% RH	0.836 ± 0.0230^a^
70 °C – 0% RH	0.856 ± 0.0341^b^
80 °C – 0% RH	0.856 ± 0.0184^a, b^
90 °C – 0% RH	0.875 ± 0.0225^b^

A similar mean value was found for R_lock_/R_0_ irrespective of drying conditions. To determine if there are statistically significant differences between the means, a one-way ANOVA across the different relative humidities and temperatures was carried out. The one-way ANOVA revealed no significant effect of relative humidity (F(3,12) = 1.022, p = 0.417) or temperature (F(2,10) = 0.788, p = 0.481) on the normalized radius at the locking point. A comparable outcome was found by Lin and Chen (2004), who dried milk droplets using air temperatures varying from 67.5 °C to 106.6 °C. For droplets with initial solids content of 30% (w/w) the change in droplet diameter per timestep was hardly affected by drying air temperature. Also, Siemons et al. (2020) found a similar trend, reporting a normalized radius of 0.82 at the moment of locking for maltodextrin droplets with DE 38, when varying drying air temperatures from 60 °C to 90 °C. Modification of the sessile single droplet drying setup, especially different contact angle and drying tunnel design probably explains why a slightly different normalized radius was found in this study. In alignment with this study, Siemons et al. (2020) observed that drying air temperature also does not influence R_lock_/R_0_ at the locking point. Based on this, it is expected that a critical solute mass fraction is required for locking of the droplet skin. Because the internal solute concentration profile is expected to vary for the different drying conditions, it may be hypothesized that the locking process is primarily determined by the mass fraction gradient close to the droplet's surface.

### 3.2 Determining stickiness through binary collisions

Different collision regimes can be experimentally determined through binary collisions. Especially the sticking regime of drying droplets is of interest as sticking collisions in spray dryers lead to the formation of agglomerates. In this section we report on the effect of relative humidity on collision regimes, which are also expected to change as a function of the drying time. The results of the collision experiments are depicted in Fig. 6. The x-axis shows the collision time, which is similar to the drying time, while the y-axis shows the collision outcome. Also, the density distribution of the collision outcomes is displayed since some outcomes are more prevalent at certain time points than others. The Gaussian KDE was constructed according to the method described in section 2.2.3.2. From Fig. 6 it becomes clear that binary collisions sequentially result in merging, sticking, and bouncing and all collision outcomes are delayed upon increasing relative humidity.

**Fig. 6:**
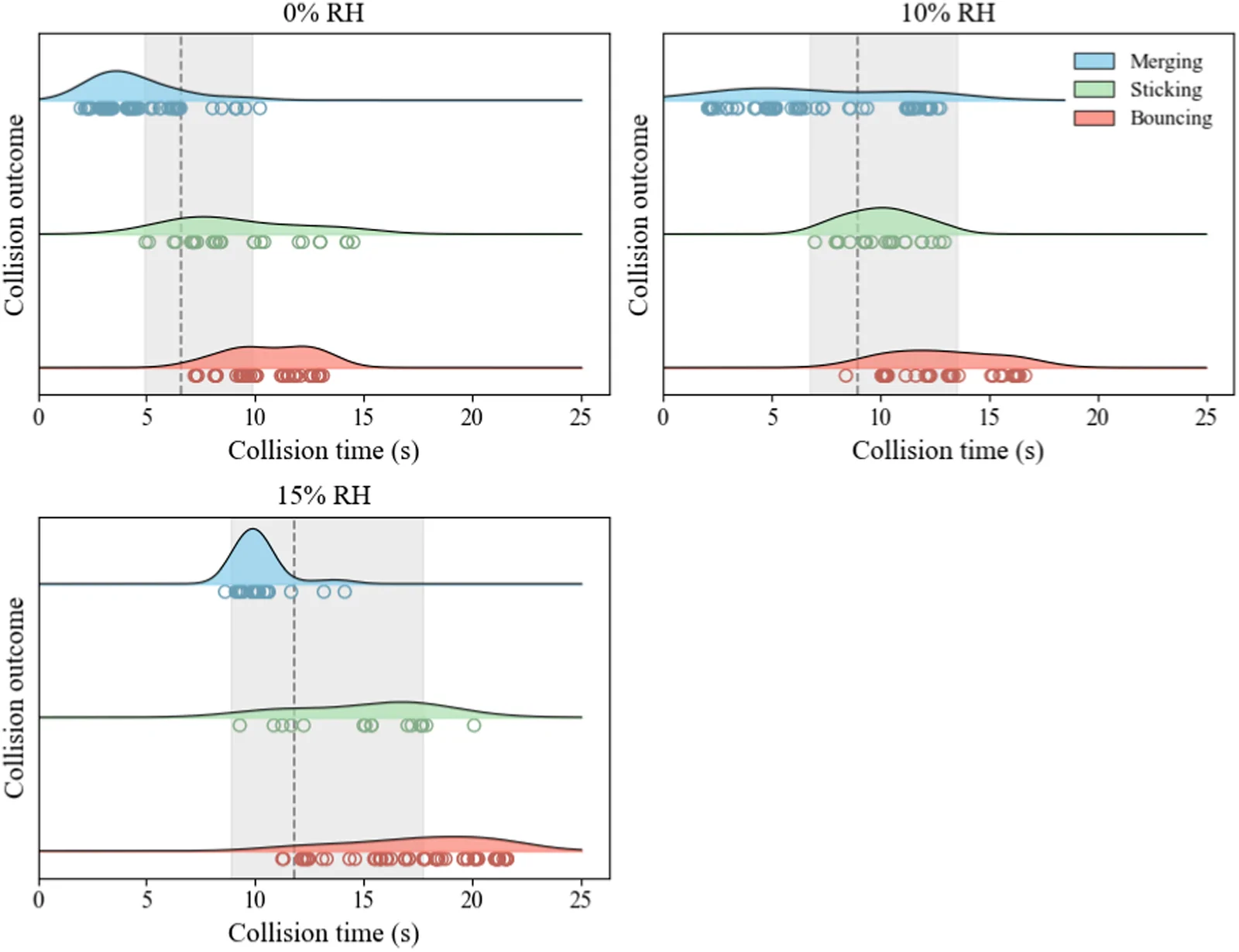
Collision outcomes over time, for MD (DE38) droplets dried using differing relative humidities. The dotted lines mark the respective locking point time. The circles indicate the observed collisions, whereas the distribution functions indicate the spreading of the observations. The grey shaded region represents the previously established stickiness regime from 0.75 to 1.5∙t_lock_.

In Fig. 6, it can be observed that all collision regimes are delayed in time when relative humidity of the drying air is increased from 0 to 15%. This was expected, since the locking point is also delayed which leads to delayed accumulation of solutes close to the surface and so slower skin formation. At t < t_lock_, collisions result in merging since the droplet has hardly dried. Bouncing collisions occur primarily after the locking point time and become more prevalent as drying continues. Focusing on the stickiness regime, previous collision studies concluded the stickiness regime occurs from approximately 0.75-1.5∙t_lock_ (de Leeuw et al., 2025; Eijkelboom et al., 2024; van Boven et al., 2024). This followed from experiments where the formulation of the drying droplet was varied, both for drying of maltodextrins with differing DE solutions and maltodextrin-protein solutions, and for collisions with different particle size and shape of the collision. Also, in this study, which focuses on the effect of relative humidity and temperature, sticking collisions are observed between 0.75∙t_lock_ and 1.5∙t_lock_, except for a few outliers. This can be observed in Fig. 6, where most sticking collisions occur within the shaded region. When considered alongside previous collision studies, the results are quite conclusive that sticking collisions center around the locking point, which highlights the importance of the locking point again. Also, applicability of such trends for multiple drying conditions is essential for studying sticking collisions that lead to agglomerate formation during spray drying. These observations form the foundation for the subsequent modeling analysis, in which the coupled mass and heat transfer of the droplet are described and connected to the sticking regime during binary collisions with single drying droplets.

### 3.3 Single droplet modeling & validation

To extrapolate the experimental findings presented above to pilot and industrial scale spray drying and to generalize to other conditions, it is of essence to be able to capture drying kinetics and in particular surface properties with a model. Therefore, a single droplet drying model was used to predict the drying behavior of the sessile droplets. The model was verified by monitoring the volume decrease until the locking point and by comparing the predicted temperature of the droplet to the experimentally observed droplet temperature.

#### 3.3.1 Droplet volume

In Fig. 7 the experimental results and the model predictions for volume decrease over time for the different drying conditions are presented. The experimental results are shown until t_lock,_ since around the locking point time mechanical instabilities, such as buckling and cracking may occur, which lead to non-ideal shrinkage (Sadek et al., 2016). At this point, the drying rate cannot be estimated by image analysis anymore. The volume decreases linearly with time during the constant drying period and as expected, the rates become less steep upon increased relative humidity and lower temperature. The model predictions align well with the experimental values till the locking point time. Only for drying at 80 °C and 15% RH and 70 °C and 0% RH, model predictions slightly deviate at the end of the initial period, which may be due to small experimental variations in surface and air temperature.

**Fig. 7:**
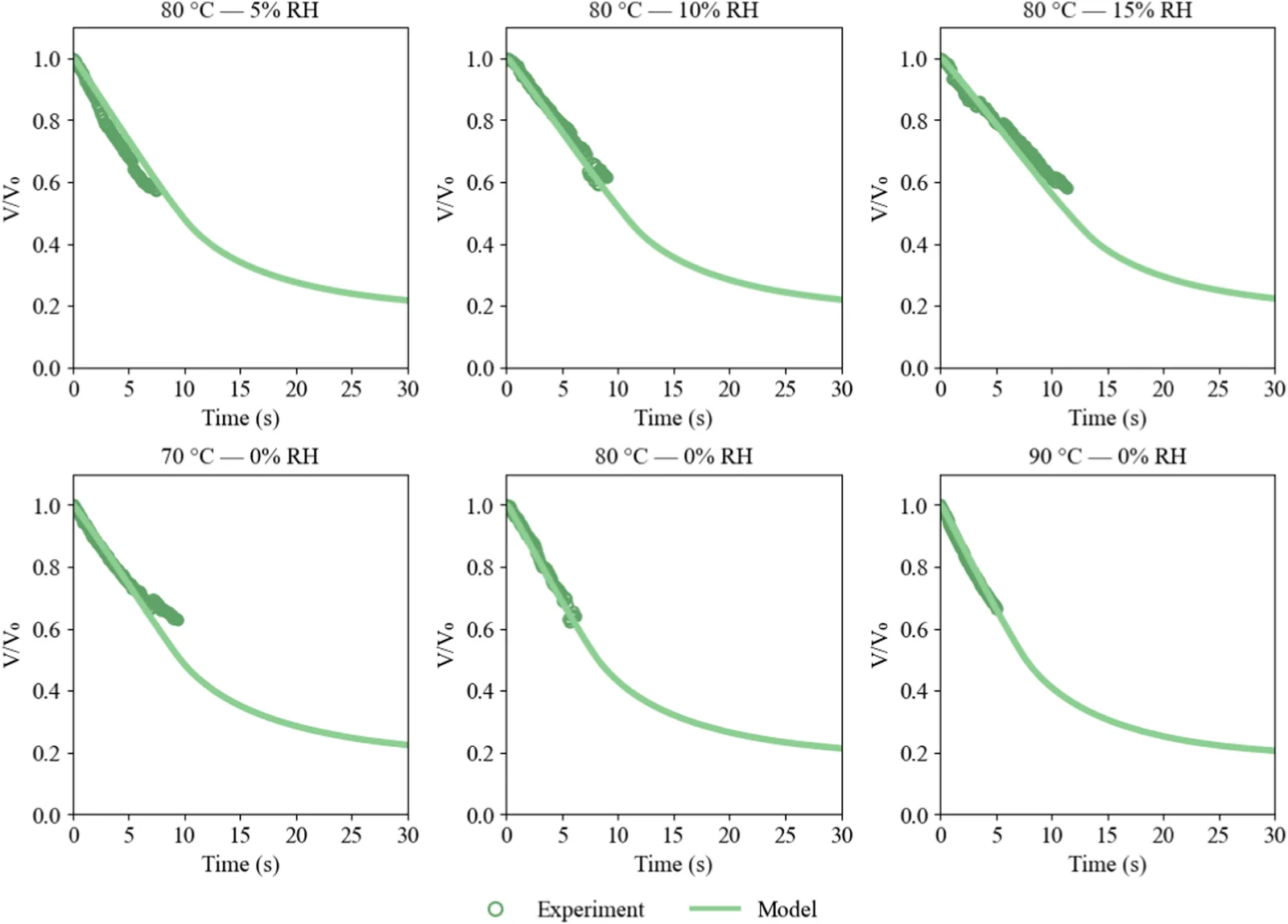
Experimental and modeled droplet volume decrease over time for drying at different relative humidities and temperatures of the drying air. Experiments were carried out in four-fold, the plotted values are average values.

#### 3.3.2 Droplet temperature

Fig. 8 shows the predicted and measured droplet temperature for drying at different relative humidities and temperatures of the drying air. Next to these temperatures, also the air and surface temperatures measured close to the droplet are shown. First, it was experimentally observed that drying droplets obtain initially a constant temperature due to evaporative cooling and thermal conduction via the surface, roughly up until the locking point time. It can be observed that this temperature increases if the relative humidity of the drying air is increased, which is caused by a decreased evaporation rate and so reduced evaporative cooling (Archer et al., 2020; Su et al., 2018). As validation, preliminary simulations were performed with $F_{conduction}=0$. If no conductive heating occurs, the constant rate temperature is equal to the wet bulb temperature (T_wb_). In line with our findings, the wet bulb temperature also increases upon increasing relative humidity and is 28, 33, 39 and 44 °C for 0, 5, 10 and 15% RH, respectively. During the subsequent falling rate period, a skin starts to form around the droplet and due to reduced evaporation, droplet temperature starts to increase until the final droplet temperature is reached, which is close to the surrounding air temperature (Grosshans et al., 2016). This final droplet temperature is not affected by the relative humidity of the drying air. Similar droplet temperature profiles were reported by Griesing et al. (2016) for levitating mannitol-water droplets dried at relative humidities varying from 1 to 15%. An increase in overall droplet temperature was observed if the drying air temperature was higher. So, at lower air temperatures slower drying resulted in delayed skin formation and the droplet resided at the lower constant temperature for an extended period of time. Eijkelboom et al., 2023a, Eijkelboom et al., 2023b and Grosshans et al. (2016) described similar results when drying single droplets using varying air temperatures of 80-120 °C and 60-90 °C, respectively.

**Fig. 8:**
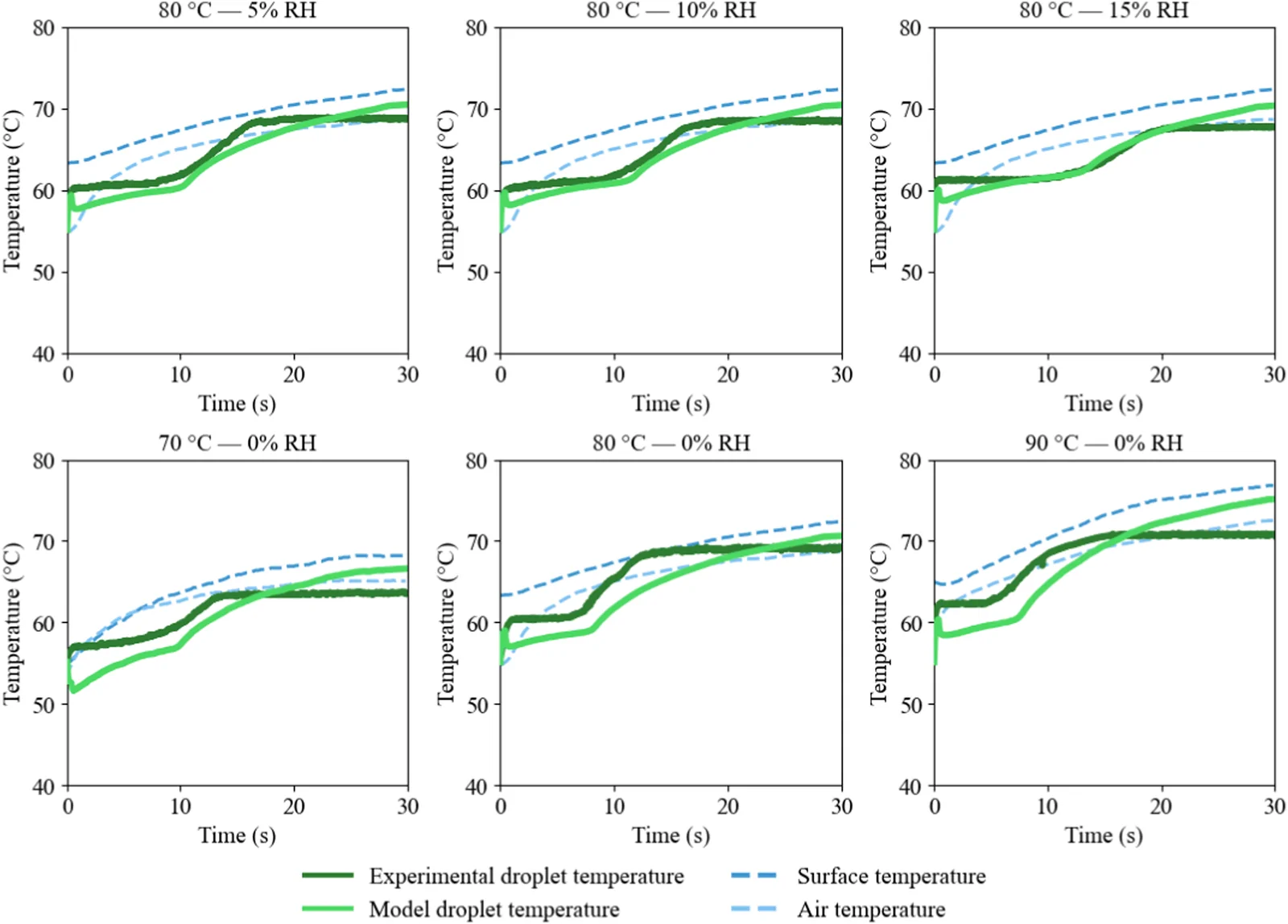
Experimental and modeled droplet temperature, as a function of time for varying RH of the drying air. Surface and air temperature are illustrated to support interpretation of the results. Experimental droplet temperatures were measured in threefold. Surface and air temperature were measured in fivefold and average values are presented in the figure. The average and standard deviation can be found in Figure A2 in the Appendix.

Although the predicted and measured droplet temperatures are not exactly similar, they show a qualitatively similar trend. Compared to the experimental results, the constant drying regime and its duration are predicted well for drying at different relative humidities. For drying at different temperatures, the predicted values are slightly lower compared to the experimentally obtained values. This may be due to an underestimation of the conductive heat transfer from the surface to the droplet in the early stages of drying leading to a lower initial droplet temperature. The onset of skin formation leading to droplet temperature increase is predicted quite well and corresponds with the expected behavior for the given RH and temperature of the drying air. Finally, the modeled droplet temperature reaches a final temperature which is closer to the surface temperature and somewhat higher than the experimentally observed droplet temperature. It is expected that conductive heat transfer is somewhat overestimated towards the end of drying. The equation for conductive heat transfer contains the contact area between the droplet and the surface which decreases as the droplet volume decreases. However, this parameter does not consider surface wrinkling which further decreases the contact area, or the decrease in heat conductivity of the droplet upon decreasing water content (Blocka et al., 2023). This would further slowdown the conductive heat transfer later in the drying process.

Combining high-resolution thermography, high speed imaging and the predictive diffusion model provides better understanding of droplet drying kinetics. The model can now be used for determining the relation between droplet properties and the stickiness regime of drying droplets, which is important for understanding stickiness and agglomeration during spray drying.

### 3.4 Model analysis of droplet properties and sticking behavior

#### 3.4.1 Critical mass fraction for locking of skin

The earlier posed hypothesis (section 3.1) suggests a critical concentration or mass fraction required to form a rigid skin causing the droplet to deviate from its spherical shape, regardless of the drying conditions. To verify this hypothesis using the developed model, the solute mass fraction profile along the radius of the droplet was calculated at the respective locking point time for varying drying air temperatures and relative humidities (from section 3.1). The results are presented in Fig. 9.

**Fig. 9:**
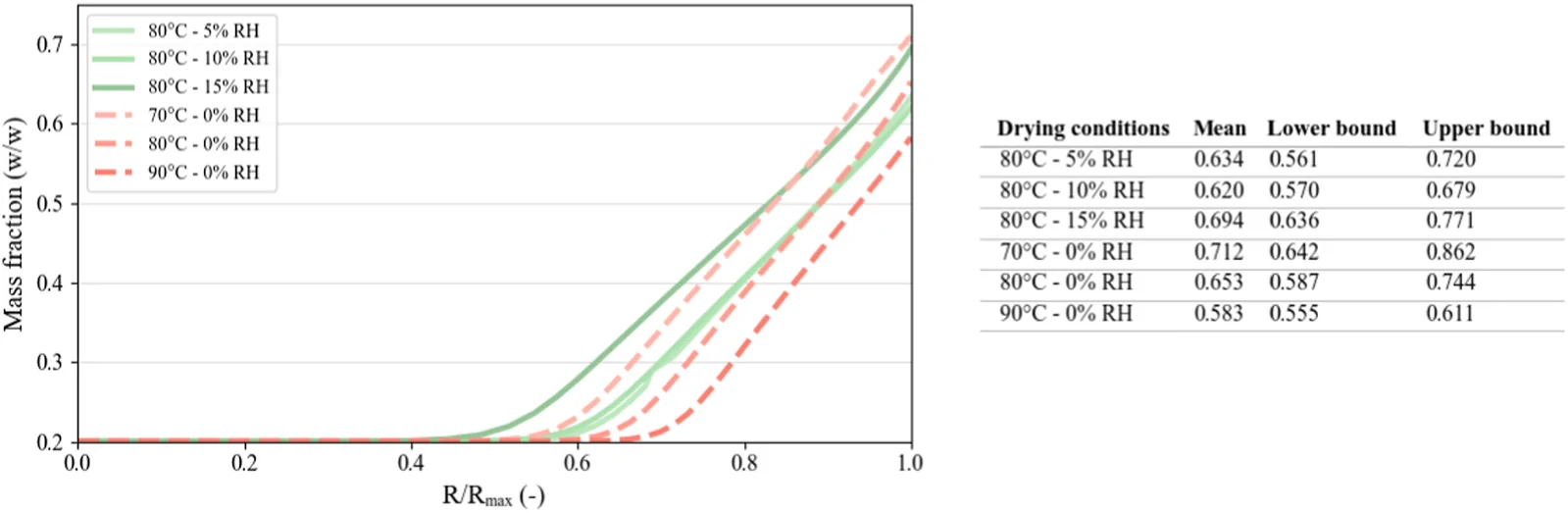
Calculated solute mass fraction profile across a normalized droplet radius (R/R_max_) at the locking point time, for varying temperatures and relative humidities for droplets with an initial radius of 200 μm. The x-axis was normalized using the maximum droplet radius at the locking point, to align the end points of the different curves. Also, it must be noted that the lines of 80 °C – 5% and 80 °C – 10% relative humidity largely overlap. The table on the right shows the mass fraction at the surface with lower and upper bound as recalculated for 95%-confidence intervals of the locking point times.

As expected, for all conditions the solute mass fraction increases towards the surface due to fast evaporation and slow solute diffusion towards the center. For drying at 80 °C and 0, 5 and 10% RH a similar mass fraction profile across the droplet was found at the locking point time. Only for drying at 80 °C and 15% RH slightly high mass fractions are observed compared to drying at other RH values and 80 °C. However, as the visual locking point time observation varies slightly, the predicted surface solute mass fraction also has some variation. This was evaluated by taking the 95%-confidence interval of the locking point time and recalculating the solute mass fractions for the boundary values of the confidence interval (Fig. 9, table). It can be observed that there is overlap between the mass fractions for all four RH conditions. The higher mass fraction for 15% RH may therefore be attributed to experimental variations in determining the locking point time. Conversely, the mass surface fraction at locking appeared not similar for different drying temperatures, especially the mass fraction close to the surface for 70 °C is higher compared that for 90 °C. Also, no overlap was found for 70 °C and 90 °C when taking the 95%-confidence interval of the locking point time into account (Fig. 9, table). This difference is hard to explain but might be due to not considering the effect of droplet temperature, which also affects the mechanical properties of the skin. The locking point time effectively marks the onset of morphology development, which can be related to the rheological properties of the skin at the locking point (Siemons et al., 2020). Rheological phenomena of polymers are, in turn, largely dependent on moisture content, which can be analyzed via glass transition temperatures (T_g_) and the actual droplet temperature (T). Therefore, analyzing the ratio of T_g_/T of the droplet surface can provide additional insights into the role of temperature and moisture content on surface properties and collision outcomes.

#### 3.4.2 Coupling collision outcomes to droplet properties

The state of the droplet skin determines to a large extent the collision outcome. For merging and sticking collisions, it can be assumed that the skin is still in the rubbery state, while for bouncing collisions the skin properties approach the glassy state (Adhikari et al., 2001; Y. H. Roos, 2002). By analyzing the ratio T_g_/T, it was aimed to relate the evolving skin properties to the different collision regimes. The ratio T_g_/T was determined for the droplet skin, where the glass transition temperature was computed with the Couchman–Karasz relation, as explained in section 2.2.4, and T is the calculated droplet temperature. The calculated T_g_/T ratios were related to the collision times and outcomes. Fig. 10 illustrates T_g_/T as a function of collision time (s), for different relative humidities and collision outcomes. Sub-graphs A, B and C include T_g_/T data of 0, 10 and 15% RH and the separate, experimentally obtained, collision outcomes are plotted on the graphs. Figure D shows the density distribution of the different collision outcomes, for all relative humidities combined.

**Fig. 10:**
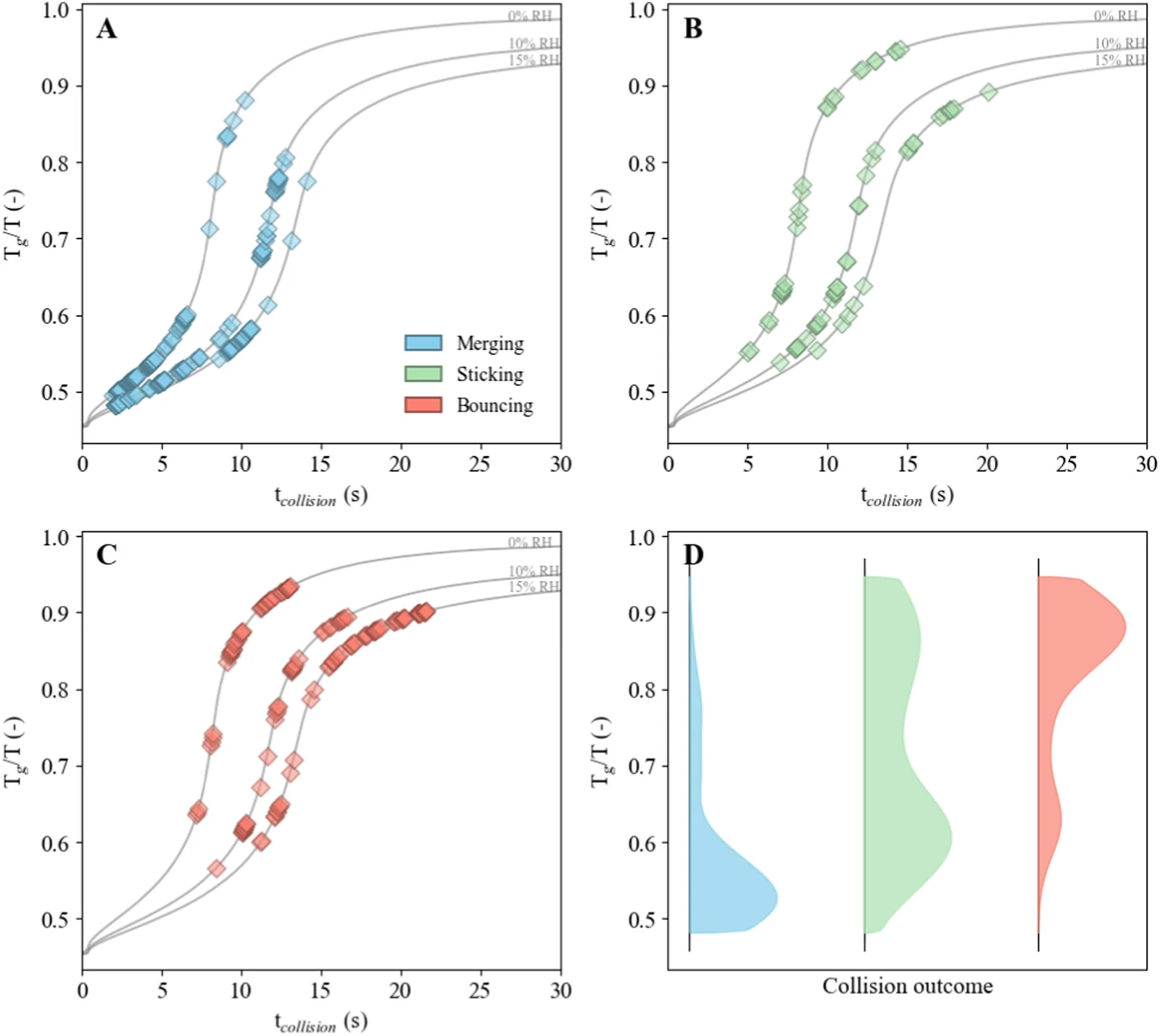
T_g_/T (−) as a function of time for relative humidities of 0, 10 and 15%. Collision outcomes are plotted on the graphs, with merging (A), sticking (B), and bouncing (C) in separate figures. Figure D shows the density distribution of collision outcomes as a function of T_g_/T.

It appeared that mostly merging occurs until a value of T_g_/T < 0.6, with some outliers particularly for drying at 10% RH. At that stage, the droplet is still most liquid-like, the droplet temperature is mostly still close to the wet bulb temperature and water is evaporating fast. Stickiness occurs at 0.55<T_g_/T < 0.9, where a higher a number of sticking collisions occurs at 0.6<T_g_/T < 0.8. Also, there is visible overlap with both merging and bouncing collisions. This was expected since variations in skin properties arise from non-uniform droplet drying, slight shifts in locking points and variability in droplet size, which inevitably contributes to variability in results. Bouncing collisions are mostly observed for T_g_/T > 0.8, where the droplet skin slowly approaches the glass transition temperature and thus obtains a glassy, rigid skin. It is expected that early bouncing collisions and late sticking collisions are potentially caused by the spatial heterogeneity of the droplets surface, caused by the non-uniform properties and relaxation of the surface (Siretanu et al., 2015). In other words, even close to glass transition, softer and stiffer patches will appear on the droplet surface, which could lead to different collision outcomes. At the same time buckling leads to formation of an irregular droplet surface, which could affect collision outcomes too.

To further generalize observed behavior across the different scenarios, time was normalized by dividing the collision time by the locking point time $\left(\frac{t_{collision}}{t_{lock}}\right)$. The results of all different drying conditions can be found in Fig. 11A. First, the overlap between the curves of 0, 5, 10 and 15% RH further adds to the validity of the single droplet drying model. Focusing on temperature, similar curves were obtained for drying at 80 and 90 °C and also greatly overlap with the different RH scenarios. Drying at 70 °C slightly deviates from the general trend. This could be due to an underestimation of the locking point, where a later locking point results in a shift of the graph towards the left. It was found at t_lock_ T_g_/T ≈ 0.6.

**Fig. 11:**
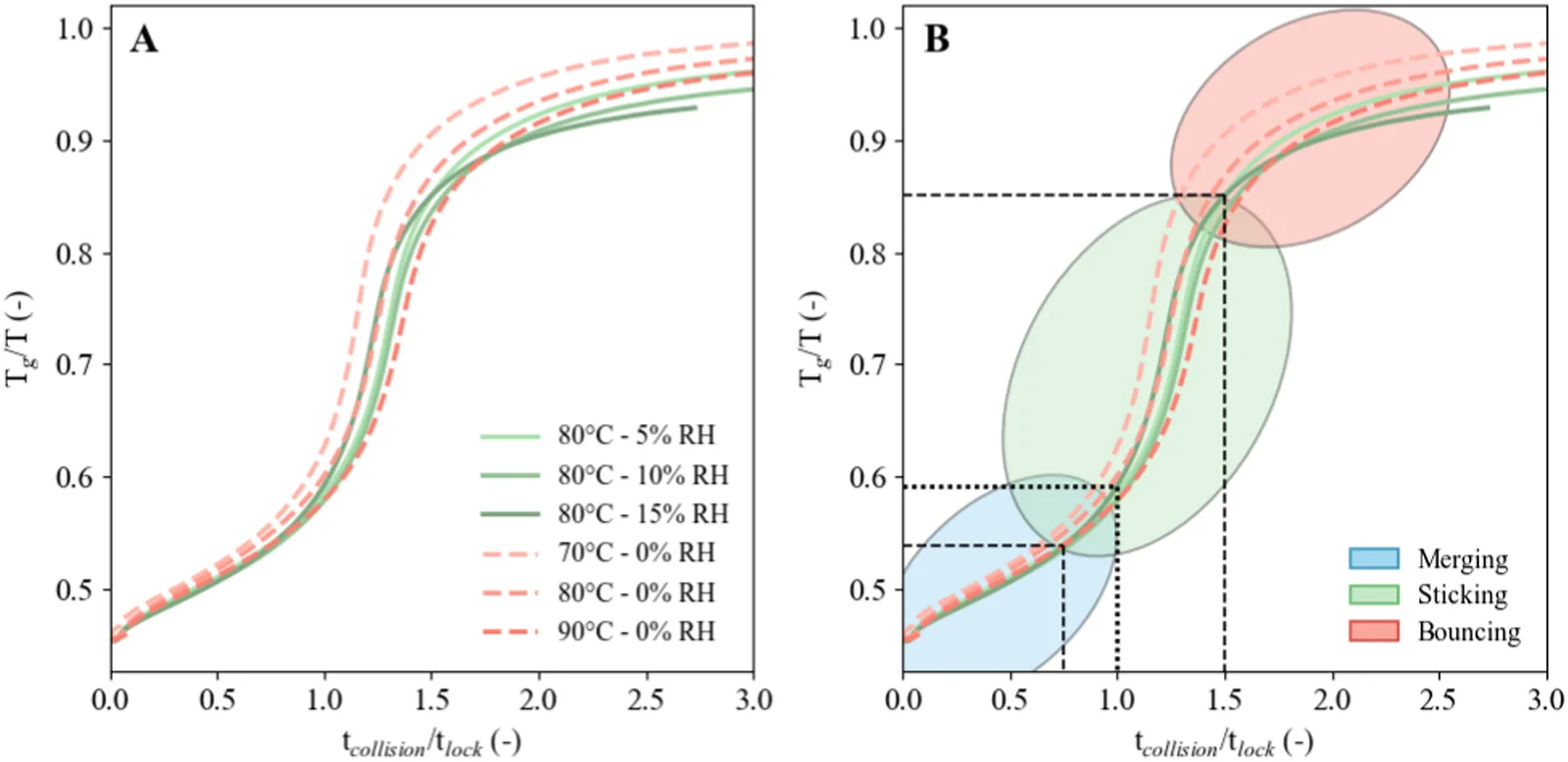
T_g_/T (−) as a function of time for different relative humidities and temperatures (A), with the expected collision regimes visualized (B). The stickiness regime is indicated using (**- -**) and the locking point time using (■■).

A projection of the different collision regimes was mapped on the curves in Fig. 11B. Since the curves overlap and almost collapse onto one curve, the results from Fig. 10 could be used to approximate the collision regimes for all drying conditions. Based on that, it was observed the stickiness regime lasts from approximately 0.75 to 1.5 times the locking point time. This has been observed in previous collision research where drying droplets consisting of maltodextrin with varying DE and indicates the state of the droplet skin is dominant in determining collision outcomes (Eijkelboom et al., 2024). Therefore, it can be concluded that a combination of experiments and modeling can be used to predict the stickiness regime of drying droplets for varying drying conditions. This method could, subsequently, help to steer agglomeration in large scale spray drying operations.

### 3.5 Predicting the stickiness regime in spray drying

In this research it was shown the stickiness regime of drying droplets can be evaluated and predicted at the single droplet scale. To steer agglomeration in spray dryer operations it is important to translate the results obtained at droplet scale to larger scale. In this section we summarize the findings of this research into a methodology that could be used for determining the stickiness regime of drying droplets in spray drying. The methodology is explained in Fig. 12.

**Fig. 12:**
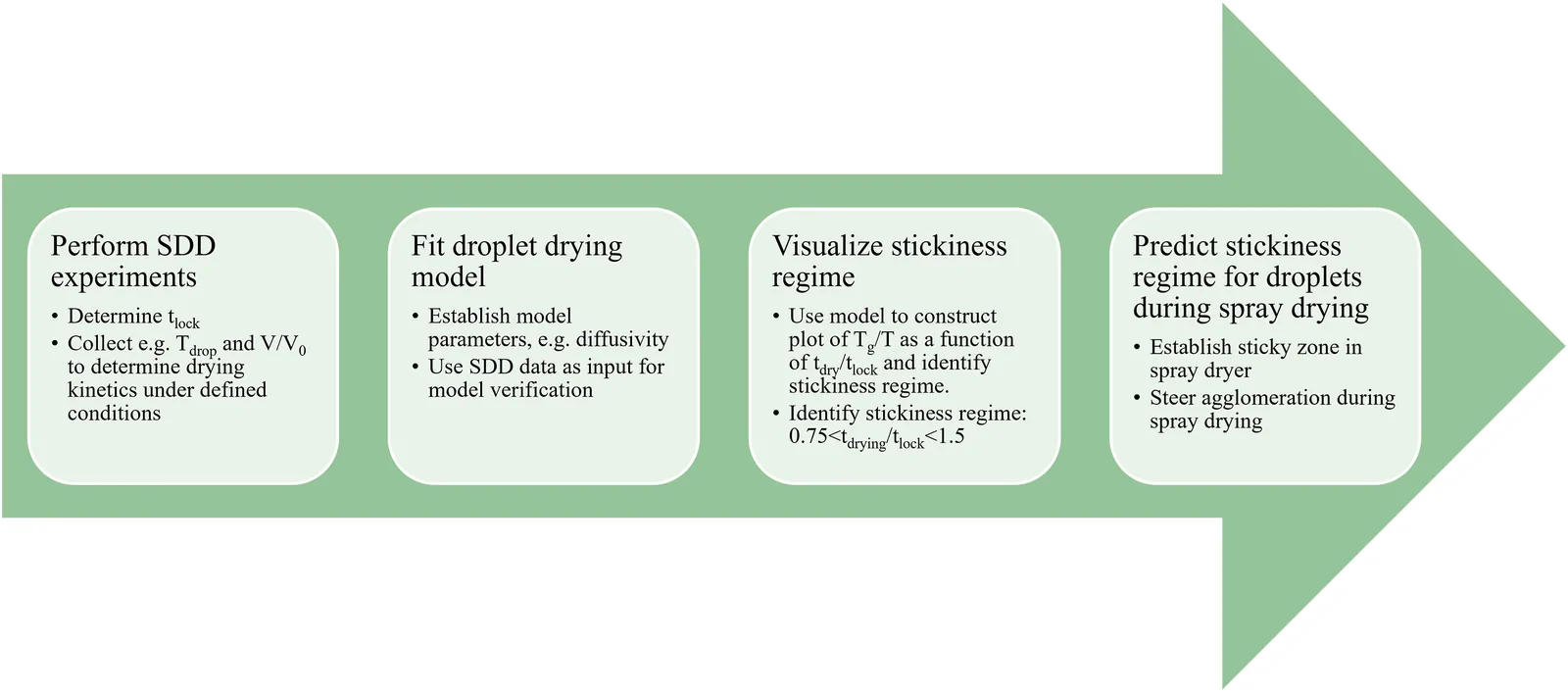
Overview of methodology proposed for determining the stickiness regime of single droplets consisting of different formulations and/or dried at different drying conditions.

According to the findings presented in this research, the stickiness regime of drying droplets can be determined using single droplet drying experiments and one-dimensional modeling. A limitation of this method may arise when assessing formulations where glass transition temperature cannot be easily determined, for example for globular proteins (Watanabe et al., 2019). At the same time droplet size should be considered, as the thickness of the skin can be important when determining T_g_/T. Also, the presented framework should be assessed for drying with higher drying air temperatures, which are even closer to mimicking spray drying conditions. At higher drying air temperatures, it becomes increasingly difficult to accurately determine the locking point using SDD approaches, since the locking point time decreases rapidly as temperature increases. The same is true for droplets with high solids concentration, which is often used in feed for spray dryers. Ultimately, determining the stickiness regime for different combinations of formulation and drying conditions should aid in further enhancing agglomeration in spray drying processes.

## 4 Conclusions

Nozzle zone agglomeration is key in determining the quality of spray dried powders. This study focused on understanding the effect of temperature and relative humidity on agglomeration, for which sticking collisions are crucial. Droplets of 20% (w/w) solutions of maltodextrin DE38 were dried at RH ranging from 0 to 15% and temperatures ranging from 70 to 90 °C. Drying behavior was monitored using high speed imaging, droplet temperature was monitored using high resolution thermography, and collision regimes were determined through binary collisions. Experimental results were used to validate the predictive droplet drying model and model output was used to predict the stickiness regime across drying conditions.

Mainly, in this research a generalized method based on SDD, and the predictive model was proposed for different ingredient formulations and drying conditions to predict the sticky behavior of droplets. The method provides valuable information to steer agglomerate formation during spray drying, which was concluded after considering the effects of moisture content and temperature. Both co-define the mechanical properties of the droplet skin, and so the ratio Tg/T was determined for the surface region for each drying condition. This ratio was plotted versus the drying time. By plotting the collision outcomes onto the graphs, a distinct distribution of the collision regimes as a function of Tg/T during drying could be observed. When making the time dimensionless (via t_dry_/t_lock_) the graphs converged into a single curve, in which the stickiness regime can be identified between 0.75 < $\left(\frac{t_{collision}}{t_{lock}}\right)$ < 1.5.

Also, the increase in locking point time for increasing RH and decrease in locking point time for increasing temperature was proportional to the change in RH or temperature of the drying air. There is no significant effect of relative humidity and temperature on the normalized radius at the locking point time. Moreover, it can be concluded that the stickiness regime is delayed, but not necessarily prolonged upon increasing RH. From thermography experiments, it became clear that the constant droplet temperature at the beginning of drying increases upon increased RH because of reduced evaporation and so evaporative cooling. Drying at higher temperatures caused an overall increase of the droplet temperature through the entire drying process. This also meant that the droplet experienced a constant temperature for a shorter amount of time, which coincided with the earlier locking point. With the experimental results, the droplet drying model could be validated for both droplet volume and droplet temperature predictions. Overall, the combined insights of high-speed imaging, high resolution thermography and predictive modeling now provides a more complete understanding of stickiness behavior. The newly proposed method allows for better predicting the stickiness regime, which contributes to better understanding and control of agglomerate formation in spray drying processes.

## Declaration of competing interest

The authors declare that they have no known competing financial interests or personal relationships that could have appeared to influence the work reported in this paper.

## Figures and Tables

**Table A1:** Closure equations used in the droplet drying model.

Name	Equation	Reference
Mutual diffusion coefficient (Darken relation)	$D_{m}=Q_{D}\cdot \left(\varphi_{w}D_{s}+\left(1-\varphi_{w}\right)\cdot D_{w}\right)$ with $\varphi_{w}=\frac{V_{w}}{V_{tot}}$ as the volume fraction of water	(Darken, 1948; Siemons et al., 2022)
Solute self-diffusion coefficient	$D_{s}=D_{s,0}\cdot \frac{\eta_{w}}{\eta_{eff}}$ with $\log\left(\frac{\eta_{eff}}{\eta_{0}}\right)=4q+11q^{2}+10q^{3}$ and $q=\frac{T_{g}}{T-0.35}$	Van Der Sman and Mauer (2019)
Diffusion coefficient of water (Free volume theory)	$D_{w}=D_{0}\cdot \exp\left(\frac{-dE}{R_{gas}T}\right)\cdot \exp\left(\frac{y_{w}\cdot V_{c,w}+\xi_{b}\cdot \left(1-y_{w}\right)\cdot V_{c,s}}{B}\right)$	(Siemons et al., 2020; Van Der Sman and Meinders, 2013; Vrentas and Vrentas, 1991)
Diffusion coefficient of solute (Generalized Stokes Einstein relation)	$D_{s,0}=\frac{k_{B}\cdot T\;}{6\pi \cdot \eta_{w}\cdot a_{0}}$ with $a_{0}$ is the hydrodynamic radius (m)	(Sutherland, 1905; Van Der Sman and Meinders, 2013)
External mass transfer coefficient	$\beta_{ext}=f_{\alpha }\frac{Sh\;D_{air}}{2R}$	(Eijkelboom et al., 2024; Siemons et al., 2022)
Alpha correction factor (film theory)	$f_{\alpha }=\frac{\ln\left(1+\frac{a_{w}p_{sat}\left(T\right)-p_{vap}}{p_{0}-p_{vap}}\right)}{\frac{a_{w}p_{sat}\left(T\right)-p_{vap}}{p_{0}-p_{vap}}}$	Adhikari et al. (2007)
Sherwood number (Ranz Marshall)	$Sh=2+0.6{Re}^{0.5}{Sc}^{0.33}=2+0.6{\left(\frac{U2R}{v_{air}}\right)}^{0.5}{\left(\frac{v_{air}}{D_{air}}\right)}^{0.33}$	(Adhikari et al., 2005; Ranz and Marshall, 1952)
Heat transfer coefficients	$h_{eff}=\frac{1}{\frac{1}{h_{\mathrm{int}}}+\frac{1}{h_{ext}}}$ with $h_{ext}=\frac{Nu\;\lambda_{air}}{H_{drop}}$ and $h_{\mathrm{int}}=\frac{\lambda_{eff}}{0.2\;r}$	Van der Sman (2003)
Nusselt number (Ranz Marshall)	$Nu=2+0.6{Re}^{0.5}\;{\mathrm{Pr}}^{0.33}=2+0.6{\left(\frac{U2R}{v_{air}}\right)}^{0.5}{\left(\frac{v_{air}}{\alpha_{air}}\right)}^{0.33}$	Adhikari et al. (2007)
Effective density	$\rho_{eff}=\frac{1}{v_{spec}}$, with $v_{spec}=\frac{1}{\frac{y_{w}}{\rho_{w}}+\frac{1-y_{w}}{\rho_{s}}}$	
Effective heat capacity	$c_{p,eff}=y_{w}c_{p,w}+\left(1-y_{w}\right)c_{p,s}$	

**Table A2:** Material specific parameters for maltodextrin DE38 and other input parameters for single droplet drying model.

Parameter	Value
Initial droplet radius (μm)	200
Molecular weight $m\left(g\cdot {mol}^{-1}\right)$	474
Viscosity $\eta_{0}\left(mPa\cdot s\right)$	0.22
Interaction parameter $x_{ws}\left(-\right)$	0.99
Initial solute mass fraction $y_{s}\left(-\right)$	0.2
Initial droplet temperature $T_{init}\left({}^{\circ}C\right)$ (average value based on all experiments)	54.2
Initial air temperature $T_{air}\left({}^{\circ}C\right)$ (average value based on all experiments)	57.0
Initial surface temperature $T_{surf}\left({}^{\circ}C\right)$ (average value based on all experiments)	61.5
Initial air velocity $U_{air}\left(m\cdot s^{-1}\right)$	0.001
Heat capacity of water $c_{p,w}$ (J/kg· K)	4200
Heat capacity of solute $c_{p,s}$ (J/kg· K)	1000
Density of water $\rho_{w}$ (kg/m^3^)	997
Density of solute $\rho_{s}$ (kg/m^3^)	1550

**Fig. A1:**
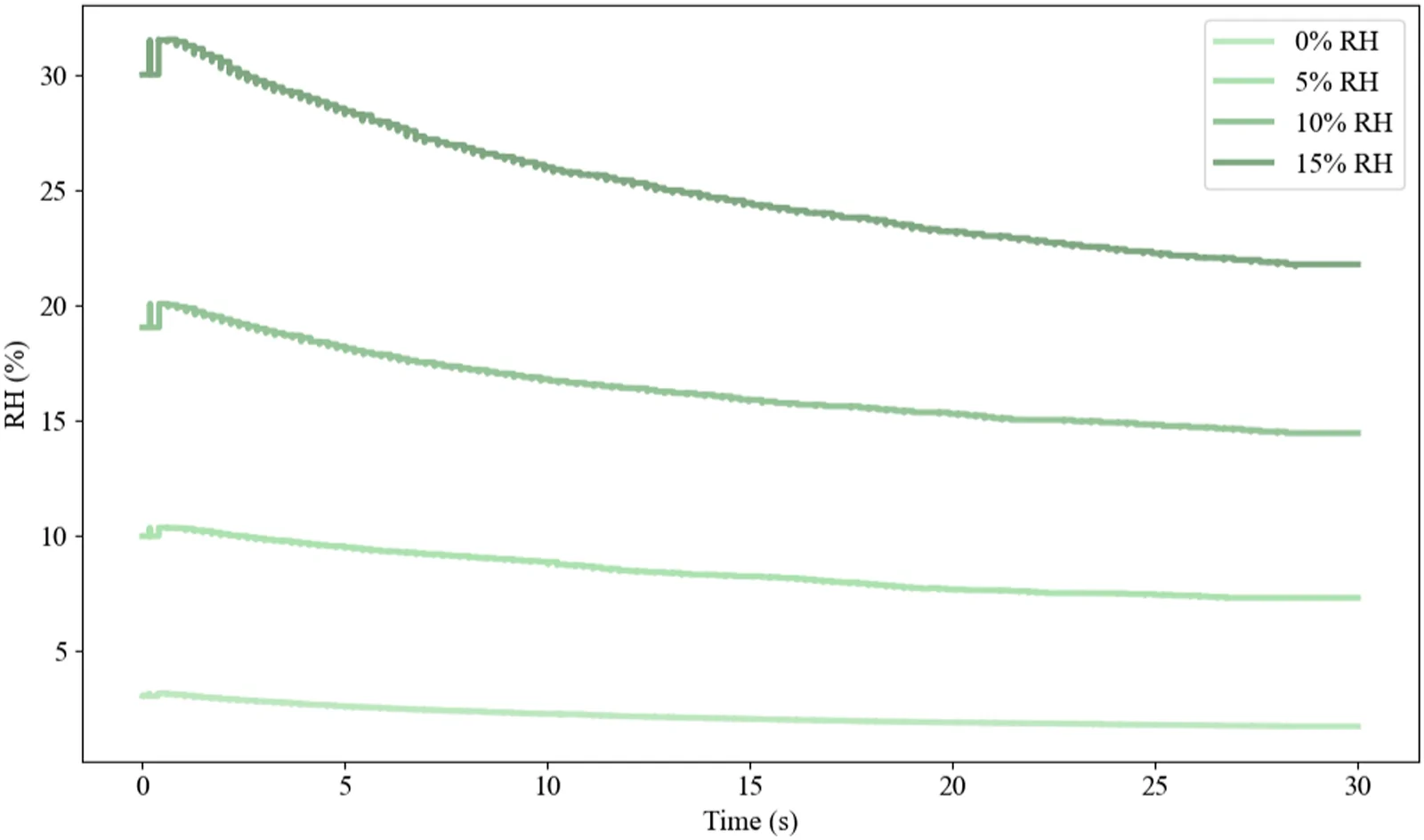
Relative humidity computed over time, at approximately 1 cm from the droplet, based on the measured air temperature, calculated saturation pressure and known mole fraction of water in the air.

**Fig. A2:**
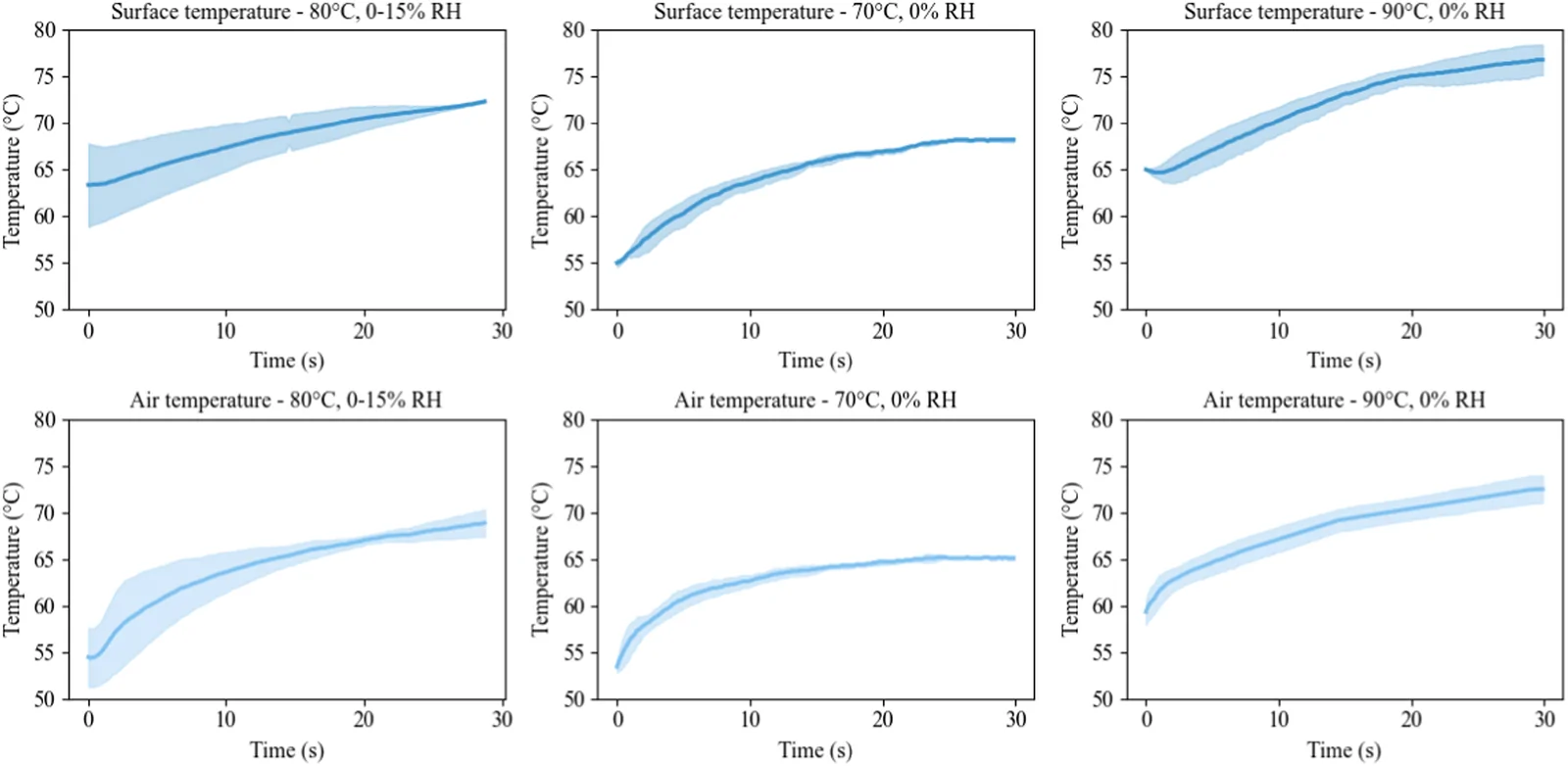
Surface and air temperature as measured close to the droplet, with the shaded area being the standard deviation of these measurements.

**Table:** 

Symbol	Definition	Unit
$A_{drop}$	Surface area of droplet	m^2^
$A_{contact}$	Contact area between droplet and surface	m^2^
$a_{w}$	Water activity	-
Bi	Biot number	-
$c_{p,eff}$	Effective heat capacity	J/kg·K
$c_{p,v}$	Specific heat capacity of vapor	J/kg·K
$c_{p,w}$	Specific heat capacity of water	J/kg·K
$c_{w}$	Mass concentration of water	kg/m^3^
$c_{w,0}$	Initial water concentration	kg/m^3^
$D_{m}$	Mutual diffusivity coefficient	m^2^/s
$f_{cap}$	Geometric correcting factor	-
$h_{cap}$	Height of the cap	m
$H_{drop}$	Height of the droplet	m
$\Delta H_{evap,0}$	Heat of vaporization	J/kg
$h_{ext}$	External heat transfer coefficient	W/m^2^·K
$L_{d}$	Characteristic length	m
$M_{w}$	Molecular weight of water	kg/mol
$m_{w,i}$	Mass of water	kg
$m_{s,i}$	Mass of solute	kg
$p_{0}$	The atmospheric pressure	Pa
$p_{vap}$	Vapor pressure	Pa
$p_{sat}$	Saturated vapor pressure	Pa
$p_{tot}$	Total pressure	Pa
r	Radial coordinate	m
R	Droplet radius	m
RH	Relative humidity	-
T	Droplet temperature	K
$T_{0}$	Reference temperature	K
$T_{air}$	Air temperature	K
$T_{drop}$	Droplet temperature	K
$T_{g}$	Glass transition temperature	K
$T_{g,s}$	Glass transition temperature of pure maltodextrin DE38	K
$T_{g,s}^{\infty }$	Glass transition temperature for a polymer with infinite chain length	K
$T_{g,w}$	Glass transition temperature of pure water	K
$T_{wb}$	Wet bulb temperature	°C
$u_{i}$	Velocity of moving mass	m/s
$x_{w}$	Mole fraction of water	-
$y_{s}$	Mass fraction of solute	-
$y_{w}$	Mass fraction of water	-
$\alpha_{res}$	Surface contact resistance	K/W
$\beta_{ext}$	External mass transfer coefficient	m/s
δ	Wall angle	Rad
$\lambda_{d}$	Droplet thermal conductivity	W/m·K
$\lambda_{eff}$	Effective thermal conductivity	W/m·K
$\rho_{eff}$	Effective density	kg/m^3^
$\rho_{mix}$	Density of the mixture	kg/m^3^

## Appendix

### 5.5.1 Use of artificial intelligence

Microsoft Copilot assisted in programming with Python for the single droplet drying model. Its role was limited to generating preliminary code drafts based on the conceptual input and providing suggestions for resolving coding errors or improving existing scripts, particularly for data visualization. All outputs were critically evaluated and modified to ensure correctness and relevance to the research objectives.

## References

[bib1] Adhikari B., Howes T., Bhandari B.R. (2007). Use of solute fixed coordinate system and method of lines for prediction of drying kinetics and surface stickiness of single droplet during convective drying. Chem. Eng. Process. Process Intensif..

[bib2] Adhikari B., Howes T., Bhandari B.R., Truong V. (2001). Stickiness in foods: a review of mechanisms and test methods (Pt. 1-33). Int. J. Food Prop..

[bib3] Adhikari B., Howes T., Lecomte D., Bhandari B.R. (2005). A glass transition temperature approach for the prediction of the surface stickiness of a drying droplet during spray drying. Powder Technol..

[bib4] Al-Khattawi A., Bayly A., Phillips A., Wilson D. (2018). The design and scale-up of spray dried particle delivery systems. Expert Opin. Drug Deliv..

[bib5] Archer J., Walker J.S., Gregson F.K.A., Hardy D.A., Reid J.P. (2020). Drying kinetics and particle formation from dilute colloidal suspensions in aerosol droplets. Langmuir.

[bib6] Bendada A., Derdouri A., Lamontagne M., Simard Y. (2004). Analysis of thermal contact resistance between polymer and mold in injection molding. Appl. Therm. Eng..

[bib7] Blocka C., Nabipoor Hassankiadeh M., Zhang L., Baik O.-D. (2023). Effects of moisture content and porosity on the thermal conductivity and volumetric specific heat capacity of pharmaceutical powders. Int. J. Pharm..

[bib8] Boel E., Koekoekx R., Dedroog S., Babkin I., Vetrano M.R., Clasen C., Van den Mooter G. (2020). Unraveling particle formation: from single droplet drying to spray drying and electrospraying. Pharmaceutics.

[bib9] Both E.M., Karlina A.M., Boom R.M., Schutyser M.A.I. (2018). Morphology development during sessile single droplet drying of mixed maltodextrin and whey protein solutions. Food Hydrocoll..

[bib10] Couchman P.R., Karasz F.E. (1978). A classical thermodynamic discussion of the effect of composition on glass-transition temperatures. Macromolecules.

[bib11] Darken L.S. (1948). Diffusion, mobility and their interrelation through free energy in binary metallic systems. Trans. Am. Inst. Min. Metall. Pet. Eng..

[bib12] de Leeuw S.W.C., Eijkelboom N.M., Hoonings K., López Rochés A., van der Sman R.G.M., Schutyser M.A.I. (2025). Exploring agglomeration: the role of particle size and shape in binary collisions. Dry. Technol..

[bib13] Eijkelboom N.M., Rang V.J., Breevaart S., Boom R.M., Wilms P.F.C., Schutyser M.A.I. (2024). Binary collisions of drying maltodextrin droplets and glass beads. J. Food Eng..

[bib14] Eijkelboom N.M., Swinkels A.C.M., de Ruiter J., Boom R.M., Wilms P.F.C., Schutyser M.A.I. (2023). High-resolution thermography and modelling allows for improved characterization of drying sessile single droplets. J. Food Eng..

[bib15] Eijkelboom N.M., van Boven A.P., Siemons I., Wilms P.F.C., Boom R.M., Kohlus R., Schutyser M.A.I. (2023). Particle structure development during spray drying from a single droplet to pilot-scale perspective. J. Food Eng..

[bib16] Fox T.G., Flory P.J. (1950). Second‐order transition temperatures and related properties of polystyrene. I. Influence of molecular weight. J. Appl. Phys..

[bib17] Fu N., Woo M.W., Chen X.D. (2012). Single droplet drying technique to study drying kinetics measurement and particle functionality: a review. Dry. Technol..

[bib18] Gianfrancesco A., Turchiuli C., Dumoulin E. (2008). Powder agglomeration during the spray-drying process: measurements of air properties. Dairy Sci. Technol..

[bib19] Giorgiutti-Dauphiné F., Pauchard L. (2013). Direct observation of concentration profiles induced by drying of a 2D colloidal dispersion drop. J. Colloid Interface Sci..

[bib20] Griesing M., Grosshans H., Hellwig T., Sedelmayer R., Gopireddy S.R., Pauer W., Gutheil E., Moritz H.U. (2016). Influence of air humidity on the particle formation of single mannitol-water droplets during drying. Chem.-Ing.-Tech..

[bib21] Grosshans H., Griesing M., Mönckedieck M., Hellwig T., Walther B., Gopireddy S.R., Sedelmayer R., Pauer W., Moritz H.U., Urbanetz N.A., Gutheil E. (2016). Numerical and experimental study of the drying of bi-component droplets under various drying conditions. Int. J. Heat Mass Tran..

[bib22] Hussain F., Jaskulski M., Piatkowski M., Tsotsas E. (2022). CFD simulation of agglomeration and coalescence in spray dryer. Chem. Eng. Sci..

[bib23] Jaskulski M., Wawrzyniak P., Zbiciński I. (2015). CFD model of particle agglomeration in spray drying. Dry. Technol..

[bib24] Jubaer H., Afshar S., Mejean S., Jeantet R., Xiao J., Chen X.D., Selomulya C., Woo M.W. (2020). Computationally inexpensive simulation of agglomeration in spray drying while preserving structure related information using CFD. Powder Technol..

[bib25] Lin S.X.Q., Chen X.D. (2004). Changes in milk droplet diameter during drying under constant drying conditions investigated using the glass-filament method. Food Bioprod. Process..

[bib26] Mezhericher M., Levy A., Borde I. (2011). Modelling the morphological evolution of nanosuspension droplet in constant-rate drying stage. Chem. Eng. Sci..

[bib27] Petersen T. (2015).

[bib28] Ranz W.E., Marshall W.R. (1952). Evaporation from drops. American Institute of Chemical Engineers.

[bib30] Roos Y., Karel M. (1991). Phase transitions of mixtures of amorphous polysaccharides and sugars. Biotechnol. Prog..

[bib29] Roos Y.H. (2002). Importance of glass transition and water activity to spray drying and stability of dairy powders. Lait.

[bib31] Sadek C., Pauchard L., Schuck P., Fallourd Y., Pradeau N., Le Floch-Fouéré C., Jeantet R. (2015). Mechanical properties of milk protein skin layers after drying: understanding the mechanisms of particle formation from whey protein isolate and native phosphocaseinate. Food Hydrocoll..

[bib32] Sadek C., Schuck P., Fallourd Y., Pradeau N., Jeantet R., Le Floch-Fouéré C. (2016). Buckling and collapse during drying of a single aqueous dispersion of casein micelle droplet. Food Hydrocoll..

[bib33] Schiffter H., Lee G. (2007). Single-droplet evaporation kinetics and particle formation in an Acoustic levitator. Part 1: evaporation of water microdroplets assessed using boundary-layer and Acoustic levitation theories. J. Pharmaceut. Sci..

[bib34] Schutyser M.A.I., Perdana J., Boom R.M. (2012). Single droplet drying for optimal spray drying of enzymes and probiotics. Trends Food Sci. Technol..

[bib35] Sewalt E.J.G. (2024).

[bib36] Shamaei S., Kharaghani A., Seiiedlou S.S., Aghbashlo M., Sondej F., Tsotsas E. (2016). Drying behavior and locking point of single droplets containing functional oil. Adv. Powder Technol..

[bib37] Siemons I., Politiek R.G.A., Boom R.M., van der Sman R.G.M., Schutyser M.A.I. (2020). Dextrose equivalence of maltodextrins determines particle morphology development during single sessile droplet drying. Food Res. Int..

[bib38] Siemons I., Veser J., Boom R.M., Schutyser M.A.I., van der Sman R.G.M. (2022). Rheological behaviour of concentrated maltodextrins describes skin formation and morphology development during droplet drying. Food Hydrocoll..

[bib39] Siretanu I., Saadaoui H., Chapel J.-P., Drummond C. (2015). Spatial heterogeneity of glassy polymer films. Macromolecules.

[bib40] Su Y.Y., Miles R.E.H., Li Z.M., Reid J.P., Xu J. (2018). The evaporation kinetics of pure water droplets at varying drying rates and the use of evaporation rates to infer the gas phase relative humidity. Phys. Chem. Chem. Phys..

[bib41] Sutherland W. (1905). A dynamical theory of diffusion for non-electrolytes and the molecular mass of albumin. London, Edinburgh Dublin Phil. Mag. J. Sci..

[bib42] Tran T.T.H., Avila-Acevedo J.G., Tsotsas E. (2016). Enhanced methods for experimental investigation of single droplet drying kinetics and application to lactose/water. Dry. Technol..

[bib43] Tsapis N., Dufresne E.R., Sinha S.S., Riera C.S., Hutchinson J.W., Mahadevan L., Weitz D.A. (2005). Onset of buckling in drying droplets of colloidal suspensions. Phys. Rev. Lett..

[bib44] van Boven A.P., Eijkelboom N.M., Fentsahm K.J., Gruson M.J., Boom R.M., Wilms P.F.C., Kohlus R., Schutyser M.A.I. (2024). A multiscale investigation on protein addition toward steering agglomeration and yield in spray drying. Lebensm. Wiss. Technol..

[bib50] van der Sman R., Renzetti S., Voudouris P., Asghari A., Khalloufi S. (2026). Correlation of T2 NMR with Tg/T and viscosity. Curr. Res. Food Sci..

[bib45] van der Sman R.G.M. (2003). Simple model for estimating heat and mass transfer in regular-shaped foods. J. Food Eng..

[bib46] van der Sman R.G.M., Mauer L.J. (2019). Starch gelatinization temperature in sugar and polyol solutions explained by hydrogen bond density. Food Hydrocoll..

[bib47] Van Der Sman R.G.M., Meinders M.B.J. (2011). Prediction of the state diagram of starch water mixtures using the Flory-Huggins free volume theory. Soft Matter.

[bib48] Van Der Sman R.G.M., Meinders M.B.J. (2013). Moisture diffusivity in food materials. Food Chem..

[bib49] van der Sman R.G.M., Ubbink J., Dupas-Langlet M., Kristiawan M., Siemons I. (2022). Scaling relations in rheology of concentrated starches and maltodextrins. Food Hydrocoll..

[bib51] Verdurmen R.E.M., Menn P., Ritzert J., Blei S., Nhumaio G.C.S., Sonne Sørensen T., Gunsing M., Straatsma J., Verschueren M., Sibeijn M., Schulte G., Fritsching U., Bauckhage K., Tropea C., Sommerfeld M., Watkins A.P., Yule A.J., Schønfeldt H. (2004). Simulation of agglomeration in spray drying installations: the EDECAD project. Dry. Technol..

[bib52] Veser J., van der Tuin J., Kodde J., Groot S.P.C., van der Sman R.G.M., Schutyser M.A.I. (2025). Effect of drying conditions on quality of primed cabbage seeds and ethanol degradation as promising quality parameter. Dry. Technol..

[bib53] Vrentas J.S., Vrentas C.M. (1991). Sorption in glassy polymers. Macromolecules.

[bib54] Watanabe K., Kawai K., Nagoe A., Suzuki T., Oguni M. (2019). Multiple glass-transitions of globular protein BSA aqueous solutions depending on the hydration degree. Bull. Chem. Soc. Jpn..

[bib55] Whittaker E.T. (1935). On Gauss' theorem and the concept of mass in general relativity. Proceedings of the Royal Society of London. A. Mathematical and Physical Sciences.

